# Immune Subversion and Quorum-Sensing Shape the Variation in Infectious Dose among Bacterial Pathogens

**DOI:** 10.1371/journal.ppat.1002503

**Published:** 2012-02-02

**Authors:** João Alves Gama, Sophie S. Abby, Sara Vieira-Silva, Francisco Dionisio, Eduardo P. C. Rocha

**Affiliations:** 1 Centro de Biologia Ambiental and Departamento de Biologia Vegetal, Faculdade de Ciências da Universidade de Lisboa, Lisboa, Portugal; 2 Instituto Gulbenkian de Ciência, Oeiras, Portugal; 3 Institut Pasteur, Microbial Evolutionary Genomics, Département Génomes et Génétique, Paris, France; 4 CNRS, URA2171, Paris, France; Children's Hospital Boston, United States of America

## Abstract

Many studies have been devoted to understand the mechanisms used by pathogenic bacteria to exploit human hosts. These mechanisms are very diverse in the detail, but share commonalities whose quantification should enlighten the evolution of virulence from both a molecular and an ecological perspective. We mined the literature for experimental data on infectious dose of bacterial pathogens in humans (ID50) and also for traits with which ID50 might be associated. These compilations were checked and complemented with genome analyses. We observed that ID50 varies in a continuous way by over 10 orders of magnitude. Low ID50 values are very strongly associated with the capacity of the bacteria to kill professional phagocytes or to survive in the intracellular milieu of these cells. Inversely, high ID50 values are associated with motile and fast-growing bacteria that use quorum-sensing based regulation of virulence factors expression. Infectious dose is not associated with genome size and shows insignificant phylogenetic inertia, in line with frequent virulence shifts associated with the horizontal gene transfer of a small number of virulence factors. Contrary to previous proposals, infectious dose shows little dependence on contact-dependent secretion systems and on the natural route of exposure. When all variables are combined, immune subversion and quorum-sensing are sufficient to explain two thirds of the variance in infectious dose. Our results show the key role of immune subversion in effective human infection by small bacterial populations. They also suggest that cooperative processes might be important for successful infection by bacteria with high ID50. Our results suggest that trade-offs between selection for population growth-related traits and selection for the ability to subvert the immune system shape bacterial infectiousness. Understanding these trade-offs provides guidelines to study the evolution of virulence and in particular the micro-evolutionary paths of emerging pathogens.

## Author Summary

Every pathogen is unique and uses distinctive combinations of specific mechanisms to exploit the human host. Yet, several common themes in the ways pathogens use these mechanisms can be found among distantly related bacteria. The understanding of these common themes provides useful concepts and uncovers important principles in pathogenesis. Here, we have made a cross-species analysis of traits thought to be relevant for virulence of bacterial pathogens. We have found that the infectious dose of pathogens is much lower when they are able to kill professional phagocytes of the immune system or to survive in the intracellular milieu of these cells. On the other hand, bacteria requiring higher infectious dose are more likely to be motile, fast-growing and regulate the expression of virulence factors when the population quorum is high enough to be effective in starting an infection. This suggests that infectious dose results from a trade-off between selection for fast coordinated growth and the ability to subvert the immune system. This trade-off may underlie other traits such as the ability of a pathogen to live outside the association from a host. Understanding the patterns shaping infectious dose will facilitate the prediction of evolutionary paths of emerging pathogens.

## Introduction

Bacteria are a significant part of the human body, often establishing commensal or mutualistic interactions with it [1]. Yet, some species, or some strains within species, have a significant negative impact on the host while exploiting its resources. Such antagonistic associations lead to co-evolution between the two sides, often in the form of an arms race [2]. Pathogenic bacteria aim at exploiting the host, which usually involves eluding its defenses. The known mechanisms for immune evasion are varied and sophisticated, but nevertheless a few common themes emerge that are shared between phylogenetically distant bacteria (reviewed in [3]–[6]). Passive mechanisms of immune evasion include invading immune-privileged locations, antigenic variation, development of quiescent states, and modification of the cellular envelope. Protection from the immune system is even more effective when bacteria are able to subvert the immune system. Bacteria that are able to kill professional phagocytes or to survive/replicate in the intracellular milieu of these cells are mentioned throughout this text as being able to “kill or subvert professional phagocytes” or just able to do “immune subversion” (see also Discussion for possible limitations and extensions of this definition). Such bacteria may not search to escape the immune response but rather to stimulate it. This can be advantageous if the bacteria can grow inside phagocytes or if the immune response competitively disadvantages neighboring bacteria [7], [8]. Effective subversion of immune cells involves a variety of mechanisms including induction of stress response to escape reactive oxygen species, subversion of signaling pathways, inhibition of fusion between phagosomes and lysosomes, escape into cytoplasm, production of toxin-killing phagocytes and manipulation of apoptosis [9], [10]. Knowledge of the mechanisms involved in subverting the immune system is important to effectively control virulence in clinical settings, but precise identification of the common themes behind them is essential to understand the evolution of virulence and its mechanisms [11]–[13].

Commonalities among strategies used by bacteria to subvert the host's immune system suggest the existence of trade-offs shaping the pathogens' life-histories [14], [15]. Notably, investment in increasing transmission between hosts often leads to increased virulence at the cost of faster clearance (or host death) [16], [17]. Investment in the control of the host immune system most often comes at the cost of less efficient extraction of host resources for growth and transmission [18]. As a result, the forms of the interaction of the pathogen with the immune system, i.e. its efficiency, its cost and its mechanisms, are key parameters shaping the evolutionary dynamics of the pathogen-host association. One influential categorization of virulence themes separates frontal-attack from stealth pathogens, in an analogy with classical military strategies [19]. Frontal-attack pathogens would have limited capacity to deal with the immune system, especially its adaptive component, producing acute diseases by growing fast and by secreting extracellular toxins that contribute to overwhelm momentarily the host defenses. Stealth pathogens, on the other hand, would manipulate efficiently the immune system achieving more stable associations where slow growth is compensated by increased persistence. Naturally, there is a gradient between stealth and frontal-attack strategies resulting from the use of a multitude of combinations of a variety of mechanisms.

The positioning of a bacterial pathogen in terms of virulence strategies results from the diverse life-history trade-offs shaping its evolution. To understand these trade-offs one must be able to categorize virulence mechanisms and virulence strategies. This requires empirical analysis of the association between their infectivity and other physiological and genetic traits such as the mechanisms of immune subversion, effector secretion, growth rates, motility or genome size. The mechanisms used to manipulate host immune responses are expected to be key determinants of the size of the infectious dose required to start an infection. Accordingly, it has been proposed that bacteria coding for secretion systems injecting effectors directly into host cells using type 3 or type 4 secretion systems (resp. T3SS and T4SS) are associated with low infectious dose [20]. Direct secretion allows local manipulation of eukaryotic cells by a small number of bacteria. On the other hand, extracellular effectors provided by other secretion systems would have a global action requiring many cooperating bacterial cells to secrete enough molecules to be effective [20]. Since the size of the bacterial population is determinant in the success of infection, many pathogens show quorum-sensing regulated expression of virulence factors such as toxins and adhesins [21], [22]. In the same line, fast growth is expected to contribute much more to the success of frontal-attack than that of stealth pathogens. Additionally, bacterial motility facilitates dispersion and colonization [23]–[25] and allows counteracting peristalsis and mucus flow [26]. But motility appendages also pose problems: motility facilitates phagocytosis [27], flagella are costly [28], poorly adapted to intracellular environments, where bacteria tend to use actin-based motility [29], and flagellin-mediated activation of dendritic cells is rapid and highly deleterious to bacterial survival [30]. This suggests that flagella loss might be adaptive in pathogens with little need of motility, but strongly selecting for immune evasion. All these hypotheses are consistent with theoretical arguments and can be illustrated with examples. Yet, we know of no comparative study making a statistical assessment of the association of these variables with infectious dose.

It has been argued that to establish a theory for the evolution of virulence aiming at explaining differences and similarities between pathogens one has to bridge the gap between evolutionary ecology concepts and the mechanistic processes of virulence [11], [14], [15], [31]. In this study we only analyze bacterial pathogens. Bacteria use very diverse mechanisms of pathogenicity for which there is a large accumulated body of literature. Instead of attempting to analyze directly the trade-offs shaping the virulence strategies, which is difficult even for a single pathogen, we will use a statistical approach, based on the comparative method, to assess the associations between eight variables thought to have key roles in virulence strategies. (i) Infectious dose. (ii) The ability of the bacteria to kill professional phagocytes or to survive in the intracellular milieu of these cells. (iii) The minimum generation time, i.e. the lineage's ability to grow very fast whenever conditions are favorable. (iv) The use of quorum-sensing to regulate the expression of virulence factors. (v) Bacterial motility. (vi) The use of secretion systems able to deliver protein effectors directly into eukaryotic cells. (vii) The genome size, which correlates to the size of the functional repertoire of a bacterium. (viii) The route of exposure. Our immediate objective was to clarify the factors relevant for the understanding of variation in ID50 among bacteria. Our final goal was to complement current approaches, mostly focused on how a population of parasites evolves in relation to virulence, by way of an analysis focusing on the common trends observed in a very diverse panel of bacterial pathogens.

## Results

### Variation in infectious dose is independent of genome size, phylogenetic structure and little affected by route of exposure

**Figure 1:**
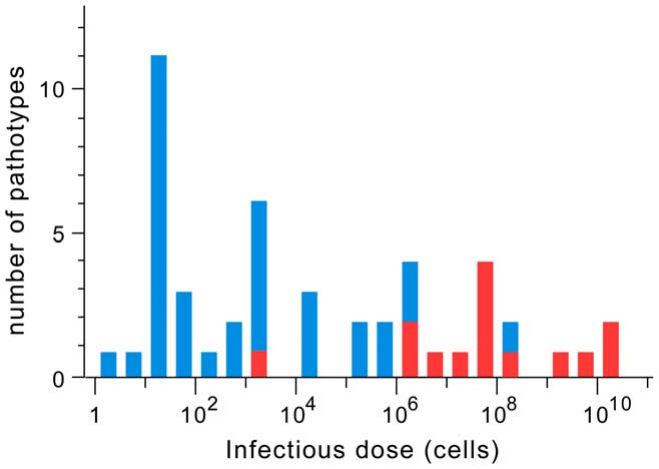
Histogram of the distribution of infectious dose values (ID50). Red (blue) represents bacteria unable (resp. able) to kill or subvert professional phagocytes.

ID50 measures the minimum size of a population of infectious agents required to start an infection with 50% probability. We computed log-transformed average values of ID50 for all 48 bacterial pathotypes for which we could collect experimental values in the literature (Figure 1, Table S1 in Text S1). Infectious dose in humans may significantly differ from that of animal models. Within humans, ID50 varies with the host health state, genetic background and the route of exposure [32] (see Discussion). Therefore, we restricted our analysis to values obtained from experimental infections of healthy human individuals using natural routes of exposure (see Text S1 for details). The only exception concerns the use of ID50 data from experiments in rhesus monkeys for *Helicobacter pylori* [33]. Exclusion of this species has no effect on the conclusions of this work. The use of ID50 obtained from human hosts prevents spurious interpretations when animal models do not adequately represent the human host. We observe a wide range of ID50 values in our dataset (Figure 1), from 3 bacterial cells in *Orientia tsutsugamushi* [34] to more than 10^10^ in *Gardnerella vaginalis* [35]. We find no clear bimodal distribution separating high and low ID50 values. Bimodality of ID50 values could have arisen from perfectly dichotomic virulence strategies, e.g. stealth versus frontal [19]. Instead, our data is consistent with a continuum of ID50 values, suggesting that more complex trade-offs shape the virulence strategies of bacterial pathogens.

Genome size in bacteria is directly proportional to the number of encoded genes and therefore corresponds to its functional potential [36], [37]. In our dataset, the smallest genomes are found among obligatory pathogens whereas larger genomes are associated with facultative pathogens [38]. This could impact infectious dose. However, we found no significant correlation between genome size and ID50 (Spearman rho = 0.18, p = 0.23), or between the number of genes in genomes and ID50 (Spearman rho = 0.20, p = 0.19). This is in line with low infectious dose in bacteria with genome sizes as diverse as the *Rickettsia* (∼1 Mb) and *Burkholderia pseudomallei* (7 Mb). We will therefore ignore the variable genome size in the subsequent analyses.

We have also analyzed the association between ID50 and the route of exposure. We classed these routes into three categories: ingestion, inhalation or other routes (including intravenous and urogenital) (Table S1 in Text S1). There is a weakly significant difference in terms of ID50 between these groups (p = 0.024, Wilcoxon test; p = 0.04, ANOVA), such that bacteria that are ingested tend to have a higher ID50 (median 1 000 000 vs 1000 for inhaled and 40 for other routes). This difference is weak. Analysis using Tukey-Kramer HSD tests do not show significant differences in ID50 between pairs of classes (p>0.05), and pairwise t-tests are at the edge of statistical significance (p = 0.03). We have not found enough data to compare the ID50 of different routes of exposure for the same pathotype. Most bacteria have one single most frequent route of exposure, the one for which we collected the experimental data. As a consequence, the values of ID50 following unnatural routes are difficult to interpret in an evolutionary context. The higher ID50 among ingested bacteria, relative to the other routes is not very surprising given the effects of stomach acidity on bacterial viability [39].

**Figure 2:**
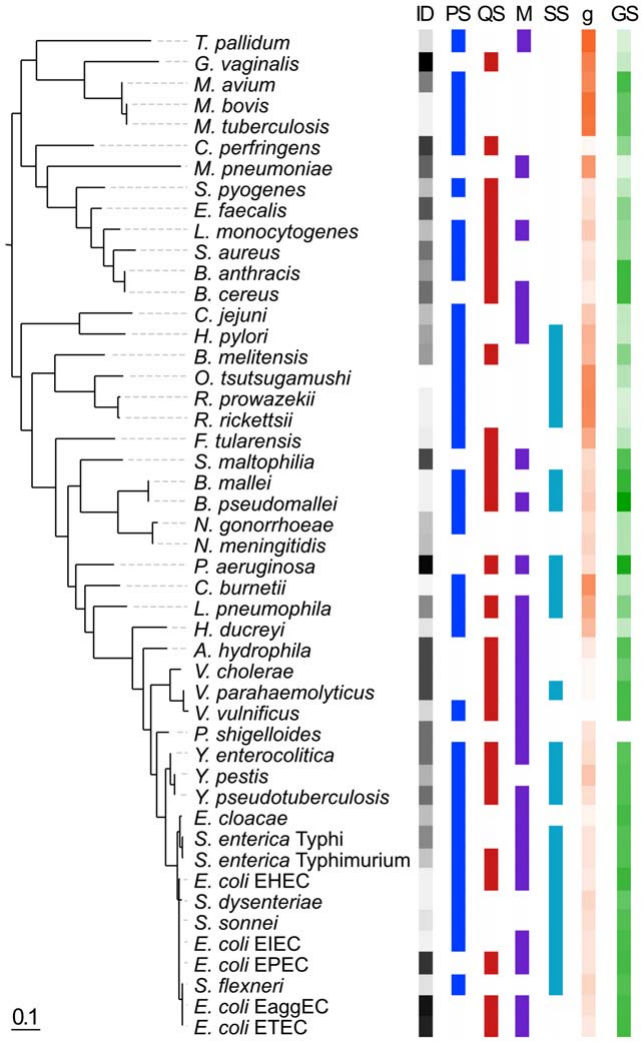
16S Phylogenetic tree linking the different pathotypes used in the work and relevant variables: ID50 (infectious dose), PS (killing of or intracellular survival in professional phagocytes), QS (virulence regulated by quorum-sensing), M (motility), SS (direct type 3 and/or type 4 secretion systems), g (minimum generation time), GS (genome size). The gradients of color from light to dark represent higher values of the quantitative variables. For discrete variables the presence of the trait is associated with dark cells and its absence with white.

Closely related strains of diverse pathotypes show large differences in ID50. An extreme case is provided by *Escherichia coli* pathotypes, including the *Shigella*, where ID50 varies between 10 and over 10^9^ cells (Table S1 in Text S1). The correlation between the value of a trait and the organismal phylogenetic relatedness is given by its phylogenetic inertia. High inertia reflects important vertical inheritance, in which case closely related organisms resemble due to their recent common ancestry [40]. We assessed the impact of phylogenetic inertia on the ID50 values, i.e. we assessed if a significant fraction of the variance in ID50 values can be explained by evolutionary relatedness. For this, we built the phylogenetic tree linking the 48 pathotypes using the 16S rRNA subunit (see Materials and Methods) (Figure 2). High phylogenetic inertia should lead to clusters of ID50 in the phylogenetic tree. Instead, using both the Blomberg's K index [41] and Pagel's Lambda index [42] we find no significant association between variation in ID50 and phylogenetic relatedness (K = 0.000394, p = 0.33; Lambda = 8×10^−05^, p = 1), indicating no need to correct ID50 statistical analyses for phylogenetic structure. These results suggest that adaptation, e.g. by horizontal gene transfer and pathoadaptive deletions, can quickly erase the signs of vertical inheritance in ID50.

### Subversion of professional phagocytes lowers infectious dose

**Figure 3:**
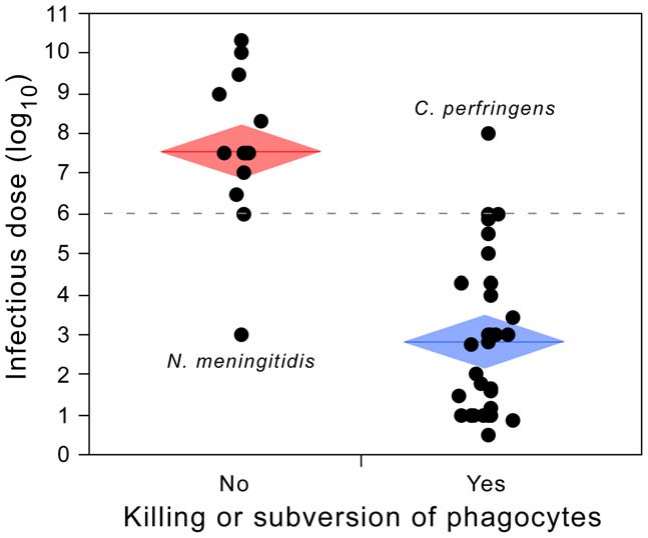
Association between infectious dose (ID50) and the capacity of pathogens of killing professional phagocytes or surviving inside professional phagocytes. Edges of the mean diamonds indicate the interval of confidence (95%) of the mean. The center of the diamond indicates the average. Outliers are labeled.

We found published experimental data indicating that 34 of the 48 pathotypes are able to kill professional phagocytes or to survive in the intracellular milieu of these cells (Table S2 in Text S1). These bacteria are expected to surmount more efficiently the immune response. We found a striking association between this trait and infectious dose (Figure 1). The set of bacteria with this trait has a median infectious dose of 250 cells versus about 30 000 000 cells for the remaining dataset (p<0.0001, Wilcoxon test) (Figure 3). This trait alone explains 56% of all variance in ID50 (R^2^ = 0.562, p<0.0001, ANOVA). There is a clear separation of the two groups of bacteria at the ID50 threshold of ∼10^6^ bacterial cells. The two outliers are *Neisseria meningitidis* and *Clostridium perfringens*, having respectively unexpected low and high ID50 (Figure 3). Several concordant experimental works indicate that *C. perfringens* has ID50 values higher than 10^8^ cells (see Table S1 in Text S1) in spite of its production of a toxin capable of killing macrophages [43]. Relative to other species in this study, *Neisseria* cells are specifically protected from complement by capsules of sialic acid and by the action of surface glycolipid lipopolysaccharides [44]. Also, *N. meningitidis* is capable of evading the immune system by antigenic variation [45]. Hence, the unexpectedly low ID50 value of *N. meningitidis* might result from its efficient use of other means of escaping the immune system.

We then tested the robustness of the association between ID50 and immune subversion. First, we removed *Treponema pallidum*, for which literature is somewhat equivocal on its ability to kill or subvert professional phagocytes (see comments in Table S2 in Text S1). Removing this species made no qualitative difference (R^2^ = 0.558, p<0.0001, ANOVA). We then further removed from the analysis the two pathotypes able to kill professional phagocytes but for which evidence of intracellular survival is lacking (Table S2 in Text S1), obtaining similar conclusions (R^2^ = 0.560, p<0.0001, ANOVA). Overall, these results are in excellent agreement with the hypothesis that the capacity of a bacterium to manipulate the immune system is a key determinant of the population size required for infection.

### Direct delivery secretion systems are weakly associated with low infectious dose

Bacteria often use secreted proteins to subvert or kill professional phagocytes [3]. In particular, it has been suggested that direct delivery of effectors into the eukaryotic cell allows bacteria to lower their ID50 [20]. This hypothesis predicts more frequent presence of T3SS and/or T4SS in low ID50 bacteria. It does not presume of the eventual presence or absence of other secretion systems. We therefore identified non-flagellar T3SS and non-conjugative T4SS in the genomes of these pathotypes (see Materials and Methods, Table S3 in Text S1, Table S4 in Text S1). This list includes all experimentally validated systems in the pathotypes, most of which were shown experimentally to be implicated in pathogenesis. Our analysis shows weakly significant lower ID50 for bacteria using T3SS or T4SS in pathogenesis (resp. medians of 150 and 80 000, p = 0.034, Wilcoxon test, p = 0.072, ANOVA). We tested the robustness of this analysis in several independent ways. Firstly, we excluded bacteria with cellular envelopes for which no direct delivery secretion systems are known (firmicutes, tenericutes, actinobacteria). Differences among the remaining (diderm) bacteria having and lacking T3SS/T4SS in terms of ID50 are not significant (p = 0.09, Wilcoxon test, p = 0.2, ANOVA). Secondly, we included type 6 secretion systems (T6SS) in the analysis, some of which are also capable of delivering effectors into eukaryotic cells [46], [47]. Bacteria with genomes encoding at least one of the three secretion systems (T3SS, T4SS or T6SS) are not significantly different from the others in terms of ID50 (p = 0.42, Wilcoxon test, p = 0.64, ANOVA). Thirdly, we removed from the analysis the three pathotypes lacking evidence of being able to survive inside professional phagocytes (Table S2 in Text S1) and this rendered the tests scarcely more significant (p = 0.03, Wilcoxon test, p = 0.04, ANOVA). Finally, bacteria with genomes encoding T3SS or T4SS have a slightly higher probability of being able to kill or subvert professional phagocytes (p = 0.054 and p = 0.026, Fisher's exact test, resp. for all genomes and for diderms only). We conclude that the evidence is weak for an association between the use of direct delivery protein secretion systems and low ID50 values.

### High infectious dose pathogens are more often motile, grow faster and use quorum sensing

**Figure 4:**
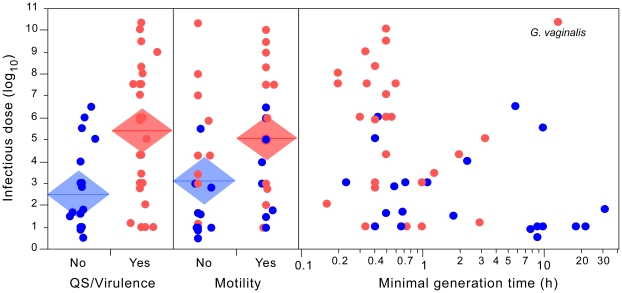
Positive association between ID50 and traits involved in cooperative growth and dispersal. We depict the association of ID50 with quorum-sensing regulated virulence (left), cell motility (center) and minimal generation time (right). Edges of the mean diamonds indicate the interval of confidence (95%) of the mean. The center of the diamond indicates the average. Outliers are labeled. The color of points depicts the values of the first variable. Blue: no information on the use of quorum-sensing to regulate the expression of virulence factors. Red: evidence for the regulation of expression of virulence factors by quorum-sensing.

The information for motility was taken from the literature [48] and checked by genome analysis (Table S4 in Text S1). Motile bacteria are associated with higher than expected values of ID50 (resp. medians 300 000 and about 150 for non-motile bacteria, p = 0.013, Wilcoxon test and p = 0.02, ANOVA, Figure 4). Motile bacteria are also less often able to kill or subvert professional phagocytes (p = 0.030, Fisher's exact test). Redoing the analysis by marking *Yersinia pseudotuberculosis* and *Listeria monocytogenes* as non-motile (Table S5 in Text S1), since they down-regulate motility when expressing virulence factors [49], [50], makes no qualitative difference (p<0.03, both for Wilcoxon test and ANOVA). Detailed statistical analysis of the effect of the differences between mechanisms of motility on ID50 was not possible because all but two bacteria in our dataset (*Mycoplasma pneumoniae* and *Haemophilus ducreyi*) use the same mechanisms of motility (flagella). Exclusion of these two bacteria did not significantly affect the association between motility and ID50 (p = 0.012, Wilcoxon test and p = 0.019, ANOVA) or the ability to kill or subvert professional phagocytes (p = 0.036, Fisher's exact test).

It has been proposed that frontal-attack pathogens compensate their high infectious dose and lower capacity to subvert the immune system by growing quickly. Fast coordinated growth could overwhelm the immune system and allow the pathogen's propagation before clearance [19], [20]. We thus tested the hypothesis that high ID50 pathogens tend to grow quickly under favorable conditions and to use quorum-sensing to regulate the expression of virulence factors. Indeed, the lowest experimentally determined minimal generation times [51](Table S5 in Text S1) are found among the pathogens with highest ID50 (Spearman's rho = −0.41, p<0.01, Figure 4). On the other hand, low ID50 bacteria include fast and slow growers. This is not unexpected since fast growers can grow slowly by way of genetic regulation whereas slow growers, by definition, never grow fast. This suggests that the capacity to grow fast is important for pathogens with high ID50 but less relevant for the others.

We then identified from the literature the bacteria with experimental evidence of using quorum-sensing to regulate the expression of virulence factors (Table S6 in Text S1). Such pathogens do have a significantly higher median infectious dose than the other bacteria (resp. 1 000 000 and 45, p<0.0005, both for Wilcoxon test and ANOVA). As expected, there is also a significant association between quorum-sensing and minimal generation times (p<0.005, both for Wilcoxon test and ANOVA) and between both variables and the ability of a pathogen to kill or subvert professional phagocytes (p<0.05, for both variables). Thus, motility, fast growth and quorum-sensing based regulation of virulence factors are associated with bacteria with high infectious dose.

### Immune subversion and quorum-sensing are major determinants of infectious dose

Many of the variables studied in this work are associated with ID50 and with each other. To disentangle their respective effects we made a forward stepwise regression analysis of the effect of these variables on ID50 (same results for mixed and backward stepwise regressions). The ability of a pathogen to kill or subvert professional phagocytes is the major explanatory variable of infectious dose (R^2^ = 0.562, p<0.0001), followed by quorum-sensing based regulation of virulence factors (+10%, cumulated R^2^ = 0.657, p<0.001). This shows that ∼66% of the variance in the ID50 values of our dataset can be explained by just two variables. The other variables have no further significant contribution to the regression (using both F-test or Akaike's criterion [52], Table 1). Hence, immune subversion and quorum-sensing are sufficient to explain all variance that can be explained by our set of variables.

**Table 1:** Results of the full and stepwise multiple regressions of ID50 with the other variables.

Variable	individual R^2^	cumulative R^2^	Prob>F	AIC
Subversion of professional phagocytes	0.562^++^	0.562	<0.00001	66.05
Virulence regulated by quorum-sensing	0.262^++^	0.657	0.001	56.25*
Route of exposure	0.127^+^	0.665	NS	56.66
Motility	0.109^+^	0.666	NS	58.63
Direct secretion systems (T3SS+T4SS)	0.068^NS^	0.666	NS	60.00
Minimal generation time	0.106^+^	0.670	NS	61.30

Variables are ordered by cumulative explanatory power in the stepwise regression from highest (top) to lowest (bottom). Both the R^2^ of the individual regressions (individual R^2^) and the cumulative effect of the variables on the stepwise regression (cumulative R^2^) are provided. For the latter, the statistical tests for the relevance of the inclusion of each variable in the stepwise regression are also given (F-test and Akaike's criterion). Statistical thresholds: ^++^ (p<0.001), ^+^ (p<0.05), NS (p>0.05).

*minimum by the AIC criterion.

## Discussion

We used methodological approaches commonly used in evolutionary ecology and comparative genomics to quantify the association of certain virulence-related traits with infectious dose. Such empirical approaches may help bridge the gap between theory in evolutionary ecology and knowledge of the molecular mechanisms of virulence [3], [19], [31]. To test quantitatively these ideas we put together variables expected to be both relevant for the mechanisms of virulence and quantifiable in very different organisms. Biological processes linked with immune defense are complex and the precise mechanisms used by pathogens to confront it can be very diverse. As a result, finding variables that can highlight commonalities among pathogens is challenging. Here, we have focused our attention on one set of traits associated with immune subversion: the pathogen's ability to kill and/or survive and/or replicate in professional phagocytes. These traits have been assessed extensively among pathogens and are important for virulence strategies. Our simplified approach remains meaningful, since we were able to explain a significant fraction of the variance of infectious dose. Future work should aim at further including strategies such as phagocytosis avoidance, interference with opsonization, antigenic variation, induction of immunopathology or taking refuge inside non-immune cells [3], [10], [53], for which systematic cross-species data is not yet available.

The robustness of our findings depends on the quality of the underlying data. Some variables, e.g. genome size, motility, secretion systems, were quantified on the basis of extensive genome data and were in excellent concordance with the literature. Minimum generation times, quorum-sensing regulated expression of virulence factors and the capacity of bacteria to resist to professional phagocytes are all extensively studied in the literature. Naturally, we cannot exclude the possibility that future studies may lead to revision of some of these numbers. To assess the robustness of our findings we have made a number of supplementary analyses where we exclude more doubtful data points. These analyses invariably confirmed the main conclusions of this work. One further source of complications in this type of analyses results from the variation of traits within pathotypes. Lack of published data forced us in a few cases to use more than one strain of a given pathotype to gather the information on the full set of traits used in the statistical analyses. Variability between strains can add noise to the data and decrease association measures. Nevertheless, the traits with lower association measures in this study, motility, genome size, secretion systems, could be analyzed using data from both literature and genome data. The two sources of data were in general concordant suggesting the robustness of our analyses. Also, we found little variation in these traits among the genomes of the strains of the same pathotype. Estimation of accurate ID50 values for bacterial pathogens is more difficult because of a number of factors including variation in the route of exposure, host health condition and genetic background [32], [54]. To reduce these sources of variation we averaged over multiple references of ID50 and used experimental values obtained from natural exposure routes in healthy humans (see Text S1). It has been suggested that ID50 does not adequately characterize the relative hazard of pathogenic organisms in humans [55]. Our results argue strongly against this perspective. If variation in ID50 were biologically irrelevant, or its measurement too noisy, then one could not have explained 66% of its variance with two pertinent biological variables. The existence of quorum-sensing based regulation of virulence shows in itself the relevance of infectious dose for virulence strategies. For obvious ethical and technical reasons, new ID50 values in humans have become almost impossible to obtain. Therefore, it will be important to study the relation between ID50 data in humans, as used in our work, and data obtained from model organisms. This would allow extending the dataset of ID50 and broaden the scope of these analyses.

Motility is less frequently found among bacteria with low ID50 and among bacteria able to kill or subvert professional phagocytes. This is in agreement with a series of observations. *Listeria monocytogenes* and *Yersinia enterocolitica* switch-off motility when up-regulating virulence factors [49], [50]. Loss of flagella is associated with the emergence of *Bacillus anthracis* lineages within the *B. cereus* clade [56], and with immune evasion in *Pseudomonas aeruginosa* [57]. These results point to a trade-off between motility and infectiousness, whereupon low ID50 bacteria tend to be non-motile, presumably as a result of investment on immune evasion and the use of actin-based motility in intracellular environments [29]. On the other hand, high ID50 bacteria are motile, possibly because this facilitates grouping, chemotaxis, transmission and life outside the host.

We found little supporting evidence for the proposal that secretion systems capable of direct delivery of protein effectors into eukaryotic cells are important determinants of low infectious dose [20]. Protein secretion by T3SS and T4SS plays essential roles in ecological interactions of pathogens and mutualists and is widespread among proteobacterial pathogens [58]–[60]. However, many bacteria lacking such systems have low ID50 and efficiently subvert macrophages, e.g. *Mycobacterium tuberculosis* [61]. This does not necessarily contradict the idea that virulence strategies are affected by the range of secreted proteins leading to local versus global effects [20]. In fact, protein secretion in very viscous environments or inside professional phagocytes is effectively local, independently of being done by T3SS/T4SS or by secretion systems targeting proteins to the extracellular environment. While the categorization of global and local effectors might still be pertinent, our data suggests it is not directly traceable from the identification of the respective secretion system. Furthermore, many bacteria use multiple means of secretion. For example, *H. pylori* blocks antigen-dependent proliferation of T-cells and suppresses B-cell apoptosis [62]–[64], which depend respectively on the secretion of VacA into the extracellular space [65] and CagA into the eukaryotic cell by a T4SS [66]. Thus, whether the secretome acts locally or globally might be less dependent on the nature of the secretion system and more on the bacterial micro-environment and the coordinated action of the different effectors.

Selection for quorum-sensing based regulation of the expression of virulence factors among high ID50 pathogens is expected since these bacteria can only effectively infect their hosts when their quorum is above a certain threshold. Quorum-sensing may be required to the establishment of cooperative processes, e.g. productive extracellular secretion of virulence factors [67]. However, such cooperative behavior can be prone to exploitation, especially in the case of multiple infections [68], if cooperating pathotypes can be outgrown by non-cooperating pathotypes with shorter minimal generation times [69]. This might explain why high ID50 bacteria grow fast. Interestingly, we find that high ID50 bacteria are more likely to enter the human body by ingestion. Since the oral cavity and the gut are the most diverse environments of the human body, bacteria in these environments are likely to face social dilemmas more frequently, and this could place particularly strong selection on growth rates, motility and cooperation by quorum-sensing. Conversely, bacteria that are introduced by insect vectors in the body experience little competition from other pathogens, and we find that they tend to grow slowly and have low ID50. Fast growth among high infectious dose bacteria also creates the demographic conditions allowing the population to counteract rapid immune clearance, as proposed in the frontal-attack model [19]. On the other hand, investment in immune evasion carries a metabolic cost that might implicate slow growth among stealth pathogens [70]. Furthermore, persistence might be favored under slow growth [71]. This suggests a trade-off between growth rate and immune subversion that could drive the evolution of frontal-attack or stealth strategies.

The ability of bacteria to kill or subvert professional phagocytes has by far the largest explanatory power over ID50 variation in our analysis. The lack of correlation between these two traits and genome size suggests that none of it requires a large catalogue of genetic information. The lack of significant phylogenetic inertia in ID50 clearly shows the potential for rapid evolution of infectious dose, e.g. by transfer or deletion of virulence factors [72], [73]. The observed large range of ID50 values is likely to result from a conjugation of factors. Immune subversion results from mechanisms with different costs and efficacies. These mechanisms are involved in trade-offs with traits that are adaptive in non-antagonistic associations. On this line of thought, one is inclined to speculate that the development of subversion skills in obligatory pathogens allows infection at low infectious dose, at the cost of poor growth outside of the host. Facultative pathogens would require higher infectious dose because they tend to remain less competent at immune subversion. Bacteria within these latter clades often receive by horizontal transfer genes rendering them more competent at immune subversion. Occasionally, some of these lineages emerge as specialists that are apt to immune subversion. This will lead or be accompanied with the evolution of lower ID50. Examples of such lineages include the *Shigella*, *Yersinia pestis* and *Bacillus anthracis*. Such adaptive shifts lead to lower growth rates and sexual isolation [74]. These costs lead to further selection for increased competence at immune subversion because such lineages are less competitive in the original environment.

## Materials and Methods

### Genome data

Data was retrieved from RefSeq GenBank Genomes (ftp://ftp.ncbi.nih.gov/genomes/Bacteria). Annotations were retrieved from the GenBank files and pseudogenes were ignored. In most cases there was more than one genome for each pathotype and in those cases we analyzed the available genomes (Table S3 in Text S1). In general the different genomes for a given pathotype produced concordant results in which case we picked one genome randomly for the phylogenetic analysis. However, in the few cases where different strains led to different results, we just analyzed the strains used to produce the infectious dose data. When even such information was unavailable we used the type-strain. The accession numbers of the reference strain are indicated in Table S3 in Text S1.

### Identification of secretion systems and flagella

For each system we initially obtained a set of proteins known to be part of the core of the system (see Supplementary Material for details). Our references for core proteins were [75], [76] for T3SS [77], [78], for T4SS [79], for T6SS and [76] for flagella. For these core proteins, we performed all against all blast searches and psi-blast searches among genomes. The hits were then clustered by sequence similarity with MCL [80]. The resulting protein families were manually curated. We then constructed multiple sequence alignments of the families using MUSCLE [81] and subsequently manually edited the alignments using SEAVIEW [82]. For each of the protein families, we built sequence profiles with HMMER 3.0 [83]. We then used the same program to perform searches with our profiles in the genomic sequences. Sets of hits with c-val <10^−3^ and aligning at least 50% of the protein profile for a given genome were further checked for co-localization of the respective genes within a replicon. These clusters were then analyzed to pinpoint the relevant systems. T3SS and flagella were separated based on the identification of one gene specific of non-flagella T3SS (the secretin) and 3 genes specific of flagella [76]. T4SS associated with protein transport were identified apart from the conjugation systems by verifying the lack of a nearby relaxase in the genome [77], [78]. We then checked extensively that our list included all systems for which experimental evidence could be found in the literature for the pathotypes of interest, which was the case (see Supplementary Material).

### Phylogenetic analyses

We extracted one 16S sequence from one representative genome of each pathotype. The sequences were aligned with MUSCLE [81] and the alignment manually corrected. We built a phylogenetic tree using PhyML with the GTR+Γ(4) model [84]. We then applied two methods to evaluate the phylogenetic signal in the ID50 data: Blomberg's K statistic [41] and Pagel's Lambda [42]. K, Lambda and the respective p-values were computed in R using the ape package [85] and the phylosig function (http://anolis.oeb.harvard.edu/~liam/R-phylogenetics/phylosig/v0.2/phylosig.R).

### Collection of data from literature

Data on infectious dose, motility, quorum sensing and bacterial interactions with the immune system were retrieved from the literature. For motility and most traits we first followed Bergey's Manual of Systematic Bacteriology [48]. For infectious dose we started by using a few reference works [32], [34], [86]–[88] as well as the reference websites of the FDA (http://www.fda.gov/Food/FoodSafety/FoodborneIllness/FoodborneIllnessFoodbornePathogensNaturalToxins/BadBugBook/), the CDC (http://www.wadsworth.org/testing/biodefense/) and the Public Health Agency of Canada (http://www.phac-aspc.gc.ca/lab-bio/res/psds-ftss/). These were then complemented with direct searches of primary literature (see Table S1 in Text S1). All these searches were done using PubMed, the Web of Science and Google Scholar with appropriate keywords. Minimal generation time data was retrieved from the supplementary material of [51], with some new data retrieved from the primary literature. All data tables and bibliographic references are published in Supplementary Material (see Tables S1 to S6 in Text S1).

## Supporting Information

Text S1 Contains Table S1 (Infectious dose data), Table S2 (Data on capacity to kill professional phagocytes or survive/replicate inside them), Table S3 (Reference genomes in GenBank), Table S4 (Protein secretion systems data), Table S5 (Motility, minimum generation times, and genome size data), Table S6 (Data on quorum-sensing). The Text S1 also contains explanatory notes for all tables and references.
(DOC)
