# Supplementary material for: Immune Subversion and Quorum-Sensing Shape the Variation in Infectious Dose among Bacterial Pathogens
Source: PLoS Pathog. 2012 Feb 2;8(2):e1002503. doi: 10.1371/journal.ppat.1002503 (PMC3271079; doi:10.1371/journal.ppat.1002503)
Supplement: Text S1 — Contains Table S1 (Infectious dose data), Table S2 (Data on capacity to kill professional phagocytes or survive/replicate inside them), Table S3 (Reference genomes in GenBank), Table S4 (Protein secretion systems data), Table S5 (Motility, minimum generation times, and genome size data), Table S6 (Data on quorum-sensing). The Text S1 also contains explanatory notes for all tables and references. (DOC) [file ppat.1002503.s001.doc]

**Supplementary Material for the article**

***Immune subversion and quorum-sensing shape the variation in infectious dose among bacterial pathogens***

By João Alves Gama^1,2^, Sophie S Abby^3,4^, Sara Vieira-Silva^3,4^, Francisco Dionisio^1,2^, Eduardo PC Rocha^3,4,*^

^1^ Centro de Biologia Ambiental and Departamento de Biologia Vegetal, Faculdade de Ciências da Universidade de Lisboa, P-1749-016 Lisboa, Portugal,

^2^ Instituto Gulbenkian de Ciência, Apartado 14, P-2781-901 Oeiras, Portugal,

^3^ Institut Pasteur, Microbial Evolutionary Genomics, Département Génomes et Génétique, F-75015 Paris, France

^4^ CNRS, URA2171, F-75015 Paris, France

* Corresponding author : erocha@pasteur.fr

**Table of contents:**

1. Infectious dose [2](#__RefHeading___Toc184792109)

2. Survival and/or killing of professional phagocytes [5](#__RefHeading___Toc184792110)

3. Reference genomes [7](#__RefHeading___Toc184792111)

4. Protein secretion systems [9](#__RefHeading___Toc184792112)

5. Other traits [12](#__RefHeading___Toc184792113)

6. Quorum sensing [13](#__RefHeading___Toc184792114)

References [15](#__RefHeading___Toc184792115)

# 1. Infectious dose

**Methods**. Data concerns exclusively ID50 measured in human hosts. The sole exception (*H. pylori*) is explicitly indicated. References indicating only upper or lower bounds for ID50 were discarded except when they consisted of very high lower limits or very low higher limits (e.g. <30 for *H. ducreyi* or >2*10^10^ for *G. vaginalis*) in which case the imprecision does not change qualitatively the character of being a very low or very high ID50 relative to the other values. ID50 values taken from immuno-compromised patients or peculiar uptakes (e.g. oral route with antacids) were excluded. We multiplied the sources of data on infectious dose to compensate for the large variance in observed values in some pathotypes. We used the average values, which are the result of arithmetic averages over the log-transformed range values.

**Specific comments** on some special cases regarding **infectious dose**:

***Helicobacter pylori***. Exceptionally, and because human data was unaavailable, the ID50 data was taken from experiments in Rhesus monkey [1] (ID50 = 10 000). There are equivalent experiments with *H. felis* in mice giving lower ID50 (100) [2]. However, *H. felis* is a different species and mice are more distant mammalian hosts. We have therefore not used this value. It has been claimed that the values of ID50 could be lower in *H. pylori* than the above-cited value, but studies are inconclusive and the ID50 is probably dependent on the use of antacids [3].

***Legionella pneumophila***. Data from humans (ID50 = 100 000) is taken from [4] and [5]. Data from guinea pigs gives lower values (12 in [6] and 129 in [7]). Value for humans has been suggested to be lower on several grounds both theoretical [8] and epidemiological [9], but the data seems controversial [10]. Given the many differences between guinea pigs and humans we just take the human data.

***Burkholderia mallei* and *B. pseudomallei***. Because of their low infectious dose they were listed in the United States as a Select Agent and Priority Pathogen of biodefense concern by the US Centers for Disease Control and Prevention and the National Institute of Allergy and Infectious Diseases. The value (ID50 = 10) was taken from the CDC data [11]. Accordingly, in hamsters *B. pseudomallei* has a LD50<10 [12]. While the two bacterial species are very close, we have decided not to exclude any of them because their genomes are very different (>1Mb difference in size) and pathologies differ.

***Vibrio cholerae***. Sewell gives a general value of 10^8^ [13]. Yet, the analysis of the data on *V. cholerae* is complex because ID50 varies considerably with the concomitant use of antacids [14]. Here, we used the most typical way of infection, i.e. oral route without the use of antacids. Several sources suggest different ID50 for O1 and non-O1 strains. For the former (including also the O139) the values are 10^6^ [15] or 10^6^-10^11^ [16], for the others they are higher, closer to the 10^8^ mentioned by [13] and [14]. Reference [17] indicates 10^11^ without food and 10^4^-10^6^ when ingested with food. Since the genomic traits we quantify are very similar among different strains we use an interval (10^6^-10^11^) and a weighted average over the logs of all these values.

***Salmonella***. The literature suggests a very significant difference in ID50 between *S. enterica typhi* and the other non-typhoidal strains. For **non-typhoid** strains earlier ID50 values are in the range 10^5^-10^7^ [18,19,20] and Kothary cites 10^5^-10^10^[21], but there are many cases later described with ID50<1000 [21,22]. The variability seems to result from the food associated with the *Salmonella*. Ingestion of food is the major mode of transmission. The references [16] and [15] cite closely related numbers resp. 1000 and 100-1000. So, we used these two latter ranges of values that reflect expert assessment of the varying literature. Since these values correspond to some, but not all non-typhoid, we restricted this class to properties arising from *S. enterica typhimurium*. For **typhoid** strains (enteric fever by *S. enterica* typhi) the values are 100 000 for [16]. A similar value is given by [13].

Table S1- Infectious dose data. Average of the arithmetic average over the log-transformed range values. Route is the route used in the experiments for determining ID50 (Inh: Inhalation; Ing: Ingestion; Oth: Other).

| ***species/pathotype*** | ***ID50  min*** | ***ID50  max*** | ***average*** | ***route*** | ***comments/references*** |
| --- | --- | --- | --- | --- | --- |
| *Aeromonas hydrophila* | 10^7^ | 10^8^ | 7.5 | Ing | [4] |
| *Bacillus anthracis* | 8000 | 50000 | 4.3 | Inh | 8 000 to 50 000 [23]; 8 000-50 000 [16] |
| *Bacillus cereus* | 10^6^ | 10^6^ | 6 | Ing | >10^6^ [16], other sources w/values relative to grams of food not absolute values ([15]and [17]) all around 10^6^. This value might therefore be a slight underestimation. |
| *Brucella melitensis* | 8000 | 50000 | 4.3 | Inh | [23] |
| *Burkholderia mallei* | 10 | 10 | 1 | Inh | [11]. See specific comments. |
| *Burkholderia pseudomallei* | 10 | 10 | 1 | Inh | 10 in humans [11], there is concordant animal data (LD50<10 in hamsters [12], and 900 in mice [24]. See specific comments above the table. |
| *Campylobacter jejuni* | 100 | 10^6^ | 3.0 | Ing | 10^2^ -10^6^ [13], 800 [17], 400-500 [15], 500 [21]. |
| *Clostridium perfringens* | 10^8^ | 10^8^ | 8 | Ing | >10^8^ [15], 5*10^8^ to get 100% infection (an over-estimation of ID) [17] |
| *Coxiella burnetii* | 1 | 10 | 0.83 | Inh | 10 [13]; 10 [16]; 1-10 [23] |
| *Enterobacter cloacae* | 1000 | 1000 | 3 | Ing | 1000 [16] |
| *E. coli EaggEC* | 10^8^ | 10^11^ | 9.5 | Ing | [21] |
| *E. coli EHEC* | 10 | 10 | 1 | Ing | 10 [17] and [16] |
| *E. coli EIEC* | 10 | 10 | 1 | Ing | 10 [17] and [15] |
| *E. coli EPEC* | 10^6^ | 10^10^ | 8.3 | Ing | 10^6^ [17], 10^8^-10^10^ [16], 10^10^ [21] |
| *E. coli ETEC* | 10^8^ | 10^10^ | 9 | Ing | 10^8^ to 10^10^ [17], [15] and [21] |
| *Enterococcus faecalis* | 10^7^ | 10^7^ | 7 | Ing | [15] |
| *Francisella tularensis* | 10 | 50 | 1.17 | Inh | 10 [13], 10-50 [23] |
| *Gardnerella vaginalis* | 2*10^10^ | 2*10^10^ | 10.3 | Oth | >2*10^10^ [25]. While this value only gives a lower bound it leads to a very high ID50, so the lower bound value was used in the analysis. |
| *Haemophilus ducreyi* | 30 | 30 | 1.48 | Oth | <30 [16] |
| *Helicobacter pylori* | 10000 | 10000 | 4 | Ing | Data for human hosts not found in the literature. Data presented is for Rhesus monkey [1]. See specific comments. |
| *Legionella pneumophila* | 100000 | 100000 | 5 | Inh | [4], [5]. See specific comments. |
| *Listeria monocytogenes* | 1000 | 1000 | 3 | Ing | [15] and [16] |
| *Mycobacterium avium* | 10000 | 10^7^ | 5.5 | Inh | [4] |
| *M. bovis* | 10 | 10 | 1 | Inh | [16] and [13] |
| *M. tuberculosis* | 10 | 10 | 1 | Inh | [16] and [13] |
| *Mycoplasma pneumoniae* | 10^6^ | 10^7^ | 6.5 | Inh | [26] |
| *Neisseria gonorrhoeae* | 250 | 1600 | 2.8 | Oth | [27] |
| *N. meningitidis* | 1000 | 1000 | 3 | Inh | [16] |
| *Orientia tsutsugamushi* | 3 | 3 | 0.48 | Oth | [13] |
| *Plesiomonas shigelloides* | 10^6^ | 10^6^ | 6 | Ing | [15] |
| *Pseudomonas aeruginosa* | 10^10^ | 10^10^ | 10 | Ing | [4] |
| *Rickettsia prowazekii* | 10 | 10 | 1 | Oth | [16] |
| *Rickettsia rickettsii* | 10 | 10 | 1 | Oth | [16] |
| *S. enterica Typhimurium* | 100 | 1000 | 2.75 | Ing | [15] and [16] |
| *S. enterica Typhi* | 100000 | 100000 | 5 | Ing | [16] and [13] |
| *Shigella dysenteriae* | 10 | 10 | 1 | Ing | 10 [15] and [21] |
| *Shigella flexneri* | 10 | 180 | 1.63 | Ing | 10 [15] and 180 [21] |
| *Shigella sonnei* | 10 | 140 | 1.57 | Ing | 10 [15] and 140 [21] |
| *Staphylococcus aureus* | 10^5^ | 8*10^6^ | 5.87 | Oth | 2-8*10^6^ [28], 10^6^ [17], 10^5^ [15] |
| *Stenotrophomonas maltophilia* | 10^6^ | 10^9^ | 7.5 | Ing | [4] (previously called *Xanthomonas*/*Pseudomonas maltophila*) |
| *Streptococcus pyogenes* | 1000 | 1000 | 3 | Ing | [15] |
| *Treponema pallidum* | 57 | 57 | 1.76 | Oth | [13] |
| *Vibrio cholerae* | 10^6^ | 10^11^ | 7.5 | Ing | 10^6^ [16], 10^6^-10^11^ [15] and 10^8^ [13]. See specific comments. |
| *Vibrio parahaemolyticus* | 10^7^ | 10^8^ | 7.5 | Ing | 10^7^-10^8^ [16], >10^6^ [15] (last reference not used in the average calculation because it does not give an upper bound for the value). |
| *Vibrio vulnificus* | 100 | 100 | 2 | Ing | [15] |
| *Y. enterocolitica* | 10^6^ | 10^6^ | 6 | Ing | [16] |
| *Yersinia pestis* | 500 | 15000 | 3.4 | Inh | [23] |
| *Yersinia pseudotuberculosis* | 10^6^ | 10^6^ | 6 | Ing | [16] |

# 2. Survival and/or killing of professional phagocytes

**Definition**. This category includes the bacteria for which there is published evidence of their ability of intracellular survival and/or replication in professional phagocytes and/or of being able to kill professional phagocytes. As professional phagocytes, we considered neutrophils, monocytes, macrophages, dendritic cells, and mast cells, although most evidence concerns macrophages and neutrophils.

We have not included in this category antigenic variation or the use of specific mechanisms to actively prevent phagocytosis without killing the professional phagocyte. In order to be included in this category there must be some evidence of either capacity to kill the phagocyte or capacity for intracellular survival/replication.

Table S2 - Data on capacity to kill professional phagocytes or survive/replicate inside them. Values within parenthesis indicate cases that for reasons explained in the "comments" column were controlled for (i.e. complementary analysis were also done for the alternative value to check that results remain robust to change in this variable).

| ***species/pathotype*** | ***Value*** | ***Comments/references*** |
| --- | --- | --- |
| *Aeromonas hydrophila* | 0 | Thought to affect only immunocompromised patients, but now regarded as an emergent pathogen [31]. No evidence of intracellular survival. |
| *Bacillus anthracis* | 1(0) | Anthrax toxins are immunosuppressors. They induce apoptosis of macrophages and dendritic cells, evade killing by neutrophils, inhibit activation of B cells, etc [32]. No evidence of intracellular survival. |
| *Bacillus cereus* | 0 | Type strain lacks the anthrax toxins of the closely related *B. anthracis* [33].No evidence of intracellular survival. |
| *Brucella melitensis* | 1 | Interferes with maturation of dendritic cells, invades and replicates within dendritic cells and macrophages [34]. |
| *Burkholderia mallei* | 1 | Survives and multiplies inside phagocytic cells [35]. The T3SS is essential for survival in macrophages [36]. |
| *Burkh. pseudomallei* | 1 | Survives and multiplies inside phagocytic cells [35]. |
| *Campylobacter jejuni* | 1 | It has been found to survive up to 7 days inside mononuclear phagocytes [37] and induces apoptosis in monocytic cell lines [38]. |
| *Clostridium perfringens* | 1 | Exhibits strong toxin-mediated cytotoxicity toward macrophages and is able to escape phagosomes of macrophages [39]. |
| *Coxiella burnetii* | 1 | Replicates within monocytes [40]. |
| *Enterobacter cloacae* | 1 | Survives in macrophage cells and destroys phagocytes [41,42]. |
| *E. coli EaggEC* | 0 | Produces heat-stable enterotoxin 1 and other virulence factors, but no evidence found of subversion of profession phagocytes. |
| *E. coli EHEC* | 1 | Contains the Shiga toxin [43], survives within macrophages [44]. |
| *E. coli EIEC* | 1 | Invades and replicates within macrophages [43]. |
| *E. coli EPEC* | 0 | Inhibits uptake by macrophages [45], but no evidence of subversion upon phagocytosis. |
| *E. coli ETEC* | 0 | Efficiently cleared by professional phagocytes [46]. |
| *Enterococcus faecalis* | 0 | Mutants sensitive to ROS have lower lifespan in macrophages [47], unclear if resistance to ROS actually allows survival to phagocytosis. |
| *Francisella tularensis* | 1 | Capable of infecting and multiplying in a wide variety of phagocytic cells including macrophages [48,49]. |
| *Gardnerella vaginalis* | 0 | Efficiently cleared by neutrophils [50]. |
| *Haemophilus ducreyi* | 1 | It resists to phagocytosis. It produces a toxin (HdCDT) that abrogates the antigen-presenting capacity of antigen-presenting cells and induces apoptosis of dendritic cells [52]. Survives intracellularly within phagocytic cells [51]. |
| *Helicobacter pylori* | 1 | Resists phagocytosis, induces macrophage apoptosis, manipulates several paths of the immune response [53], inhibits antigen presentation [54], disrupts phagosome maturation in primary human macrophages[55]. |
| *Legionella pneumophila* | 1 | Replicates within alveolar macrophages [56,57]. |
| *Listeria monocytogenes* | 1 | Survives and multiplies in macrophages [58]. |
| *Mycobacterium avium* | 1 | Replicates in macrophages [59]. |
| *Mycobacterium bovis* | 1 | Persists and replicates in macrophages [60]. |
| *Mycob. tuberculosis* | 1 | Persists and replicates in macrophages [60], inhibits antigen presentation [61]. |
| *Mycoplasma pneumoniae* | 0 | Chronic disease, but no report of active subversion of professional phagocytes. |
| *Neisseria gonorrhoeae* | 1 | Intracellular survival in polymorphonuclear granulocytes [62]. |
| *Neisseria meningitidis* | 0 | Does extensive antigenic variation. Stimulates leukocytes and monocytes/macrophages during tissue invasion and septic shock [63].No evidence of survival or replication upon phagocytosis by professional phagocytes. |
| *Orientia tsutsugamushi* | 1 | Intracellular infection of macrophages and neutrophils [64,65]. |
| *Plesiomonas shigelloides* | 0 | Produces diarrhea, septicemia and meningitis but no evidence of subversion of professional phagocytes upon phagocytosis (as the closely related A. hydrophila). |
| *Pseudomonas aeruginosa* | 0 | Secretes proteases that degrade immune system molecules [66], but we found no published evidence of killing professional phagocytes. The T3SS inoculates ExoT and ExoS into the cytosol of host cells, resulting in the paralysis of engulfment by the phagocytic cell [67], thus allowing phagocytosis avoidance but apparently not survival after phagocytosis. |
| *Rickettsia prowazekii* | 1 | Survives and grows in macrophages [68]. |
| *Rickettsia rickettsii* | 1 | Survives and grows in macrophages [68]. |
| *S. enterica Typhimurium* | 1 | Intracellular survival and proliferation in different populations of immune cells [69,70]. |
| *Salmonella enterica Typhi* | 1 | Intracellular survival and proliferation in different populations of immune cells [70]. |
| *Shigella dysenteriae* | 1 | Survival in macrophages by rapidly inducing apoptosis [71]. |
| *Shigella flexneri* | 1 | Survival in macrophages by rapidly inducing apoptosis [71]. |
| *Shigella sonnei* | 1 | Survival in macrophages by rapidly inducing apoptosis [71]. |
| *Staphylococcus aureus* | 1 | While better known for antigenic variation and avoiding phagocytosis [30], it also kills neutrophyls upon phagocytosis [72]. |
| *Stenotrophomonas maltophilia* | 0 | Opportunistic pathogen cleared efficiently by macrophages [73]. |
| *Streptococcus pyogenes* | 1 | Resistance to phagocytosis. Intracellular survival and proliferation in macrophages that are within-host reservoirs [74]. |
| *Treponema pallidum* | 1(0) | Extensive capacity for antigenic variation [75]. Evidence for intracellular invasion [77]. Macrophages seem to be the major efector of *T. pallidum* elimination during primary infection [78]. Resists to phagocytosis by macrophages but unclear if this involves just resistance to phagocytosis or survival to phagocytosis [76]. Therefore, we marked this pathotype as "1", but showed that results remained unchanged when it is 0. |
| *Vibrio cholerae* | 0 | Toxin production [79], but no report of killing or intracellular survival in professional phagocytes upon phagocytosis. |
| *V. parahaemolyticus* | 0 | Capable of evading phagocytosis by macrophages, but no evidence of intracellular survival upon phagoctosis [80]. |
| *Vibrio vulnificus* | 1(0) | Induces apoptosis in macrophages and lymphocytes [29,81, 25]. While it is able to kill macrophages, we could find no evidence that it can survive in the intracellular milieu ("1" in main analysis, changing this value to "0" made no qualitative change to results). |
| *Yersinia enterocolitica* | 1 | Resist phagocytosis by neutrophils, survive and multiply in macrophages [82]. |
| *Yersinia pestis* | 1 | Resist phagocytosis by neutrophils, survive and multiply in macrophages [82]. |
| *Y. pseudotuberculosis* | 1 | Resist phagocytosis by neutrophils, survive and multiply in macrophages [82]. |

# 3. Reference genomes

Table S3- Reference genomes in GenBank.

| ***species/pathotype*** | ***Strains*** | ***Chromosomes accession numbers*** |
| --- | --- | --- |
| *Aeromonas hydrophila* | Aeromonas hydrophila subsp. hydrophila ATCC 7966 | NC_008570 |
| *Bacillus anthracis* | Bacillus anthracis str. Ames, str. A0248, str. Sterne, CDC684, str. CI | NC_003997, NC_012659, NC_005945, NC_012581, NC_014335 |
| *Bacillus cereus* | Bacillus cereus AH187, ATCC10987, E33L, B4264, AH820, ATCC14579, G9842, Q1, 03BB102 | NC_011658, NC_003909, NC_006274, NC_011725, NC_011773, NC_004722, NC_011772, NC_011969, NC_012472 |
| *Brucella melitensis* | Brucella melitensis bv. 1 str. 16M, biovar Abortus 2308, ATCC23457 | NC_003317, NC_003318, NC_007618, NC_007624, NC_012442, NC_012441 |
| *Burkholderia mallei* | Burkholderia mallei ATCC 23344, SAVP1, NCTC 10229, NCTC 10247 | NC_006348, NC_006349, NC_008784, NC_008785, NC_008835, NC_008836, NC_009079, NC_009080 |
| *Burkh. pseudomallei* | Burkholderia pseudomallei K96243, 1710b, 668, 1106a, MSHR346 | NC_006350, NC_006351, NC_007434, NC_007435, NC_009074, NC_009075, NC_009076, NC_009078, NC_012695 |
| *Campylobacter jejuni* | Campylobacter jejuni subsp. jejuni NCTC 11168, RM1221, subsp. jejuni 81-176, subsp. doylei 269.97, subsp. jejuni 81116 | NC_002163, NC_003912, NC_008787, NC_009707, NC_009839 |
| *Clostridium perfringens* | Clostridium perfringens str. 13, ATCC 13124, SM101 | NC_003366, NC_008261, NC_008262 |
| *Coxiella burnetii* | Coxiella burnetii RSA493, RSA331, CbuG_Q212, CbuK_Q154, Dugway 5J108-111 | NC_002971, NC_010117, NC_011527, NC_011528, NC_009727 |
| *Enterobacter cloacae* | Enterobacter cloacae subsp. cloacae ATCC 13047, Enterobacter sakazakii ATCC BAA-894, Enterobacter sp. 638 | NC_014121, NC_009778, NC_009436 |
| *E. coli EaggEC* | Escherichia coli 55989, O42 | NC_011748, FN554766 |
| *E. coli EHEC* | Escherichia coli O157:H7 str. Sakai, O157:H7 EDL933, O157:H7 str. EC4115, O157:H7 str. TW14359 | NC_002695, NC_002655, NC_011353, NC_013008 |
| *E. coli EIEC* | Escherichia coli 53638 | NZ_AAKB02000000 |
| *E. coli EPEC* | Escherichia coli E22 | AAJV00000000 |
| *E. coli ETEC* | Escherichia coli E24377A | NC_009801 |
| *Enterococcus faecalis* | Enterococcus faecalis V583 | NC_004668 |
| *Francisella tularensis* | Francisella tularensis subsp. tularensis WY96-3418, subsp. Holarctica, subsp. tularensis FSC198, subsp. holarctica OSU18, subsp. novicida U112, subsp. holarctica FTNF002-00, subsp. mediasiatica FSC147, subsp. tularensis Schu 4 | NC_009257, NC_007880, NC_008245, NC_008369, NC_008601, NC_009749, NC_010677, NC_006570 |
| *Gardnerella vaginalis* | Gardnerella vaginalis 409-05, ATCC 14019 | NC_013721, NC_014644 |
| *Haemophilus ducreyi* | Haemophilus ducreyi 35000HP | NC_002940 |
| *Helicobacter pylori* | Helicobacter pylori 26695, J99, HPAG1, Shi470 | NC_000915, NC_000921, NC_008086, NC_010698 |
| *Legionella pneumophila* | Legionella pneumophila subsp. pneumophila str. Philadelphia 1, Lens, Paris, Corby, 2300/99 Alcoy | NC_002942, NC_006369, NC_006368, NC_009494, NC_014125, |
| *Listeria monocytogenes* | Listeria monocytogenes EGD-e, str. 4b F2365, HCC23, Clip81459, 08-5578, 08-5923 | NC_003210, NC_002973, NC_011660, NC_012488, NC_013766, NC_013768 |
| *Mycobacterium avium* | Mycobacterium avium subsp. paratuberculosis K-10, 104 | NC_002944, NC_008595 |
| *Mycobacterium bovis* | Mycobacterium bovis AF2122/97, BCG, PG45 | NC_002945, NC_008769, NC_012207 |
| *Mycob. tuberculosis* | Mycobacterium tuberculosis H37Rv, CDC1551, F11, KZN1435 | NC_000962, NC_002755, NC_009565, NC_012943 |
| *Mycoplasma pneumoniae* | Mycoplasma pneumoniae M129 | NC_000912 |
| *Neisseria gonorrhoeae* | Neisseria gonorrhoeae FA 1090, NCCP11945 | NC_002946, NC_011035 |
| *Neisseria meningitidis* | Neisseria meningitidis Z2491, MC58, FAM18, 053442 | NC_003116, NC_003112, NC_008767, NC_010120 |
| *Orientia tsutsugamushi* | Orientia tsutsugamushi str. Ikeda, Boryong | NC_010793, NC_009488 |
| *Plesiomonas shigelloides* |  |  |
| *Pseudomonas aeruginosa* | Pseudomonas aeruginosa PAO1, UCBPP-PA14, PA7, LESB58 | NC_002516, NC_008463, NC_009656, NC_011770 |
| *Rickettsia prowazekii* | Rickettsia prowazekii str. Madrid E | NC_000963 |
| *Rickettsia rickettsii* | Rickettsia rickettsii str. 'Sheila Smith', Iowa | NC_009882, NC_010263 |
| *S. enterica Typhimurium* | Salmonella enterica typhimurium LT2 | NC_003197 |
| *Salmonella enterica Typhi* | Salmonella enterica Typhi str. CT18, Ty2 | NC_003198, NC_004631 |
| *Shigella dysenteriae* | Shigella dysenteriae Sd197 | NC_007606 |
| *Shigella flexneri* | Shigella flexneri 2a str. 301, 2a str. 2457T, 5 str. 8401 | NC_004337, NC_004741, NC_008258 |
| *Shigella sonnei* | Shigella sonnei Ss046 | NC_007384 |
| *Staphylococcus aureus* | Staphylococcus aureus subsp. aureus Mu50, COL, MW2, JH1 | NC_002758, NC_002951, NC_003923, NC_009632 |
| *Stenotrophomonas maltophilia* | Stenotrophomonas maltophilia K279a, R551-3 | NC_010943, NC_011071 |
| *Streptococcus pyogenes* | Streptococcus pyogenes M1 GAS, MGAS8232, SSI-1, MGAS315 | NC_002737, NC_003485, NC_004606, NC_004070 |
| *Treponema pallidum* | Treponema pallidum subsp. pallidum str. Nichols, SS14 | NC_000919, NC_010741 |
| *Vibrio cholerae* | Vibrio cholerae O1 biovar eltor str. N16961, O395, MJ-1236, M66-2 | NC_002505, NC_002506, NC_009457, NC_009456, NC_012668, NC_012667, NC_012578, NC_012580 |
| *Vibrio parahaemolyticus* | Vibrio parahaemolyticus RIMD 2210633 | NC_004603, NC_004605 |
| *Vibrio vulnificus* | Vibrio vulnificus CMCP6, YJ016, MO6-24/O | NC_004459, NC_004460, NC_005139, NC_005140, NC_014965, NC_014966 |
| *Yersinia enterocolitica* | Yersinia enterocolitica subsp. enterocolitica 8081, subsp. palearctica 105.5R(r) | NC_008800, NC_015224 |
| *Yersinia pestis* | Yersinia pestis CO92, KIM 10, biovar Microtus str. 91001, Antiqua | NC_003143, NC_004088, NC_005810, NC_008150 |
| *Yersinia pseudotuberculosis* | Yersinia pseudotuberculosis IP 32953, IP 31758, YPIII, PB1/+ | NC_006155, NC_009708, NC_010465, NC_010634 |

# 4. Protein secretion systems

**Identification of T4SS**. T4SS were identified in genomes as described in detail elsewhere [83]. We used 40 profiles of proteins involved in conjugation to identify T4SS, relaxases and type 4 coupling proteins in the genomes using HMMER 3.0 with a local alignment covering at least 50% of the profile and a c-value of at least 0.001 [84]. T4SS loci dedicated to transfer of protein effectors in bacterial pathogens have a T4SS and (eventually) a T4CP, but they lack relaxases [85], although sometimes they can use relaxases in trans (e.g. [57]). On the other hand, self-conjugation of mobile genetic elements requires the presence of a relaxase [86]. One other major distinction between T4SS for protein secretion and for conjugation is the rate of gain and loss of the loci in lineages. This rate is low, i.e. the locus is stable, in the former and high in the latter. Hence, when all or nearly all genomes of a species or pathotype encoded a T4SS but no relaxase at a given locus they were classed as having a direct delivery protein T4SS. All bacteria with genomes encoding T4SS experimentally found to be relevant for virulence within our list were thus successfully retrieved, e.g. *Brucella melitensis* [87], *Coxiella burnetti* [88], *Helicobacter pylori* [89], *Legionella pneumophila* [57] or the Rickettsiales [90].

**Identification of T6SS**. Initially, we identified T6SS using a recent extensive analysis [91]. Since this reference lacked information on some of the most recent genomes, we extracted T6SS gene clusters (genomic positions according to [91]) from model genomes for which experimental validation was found in the literature. We sampled T6SS among the five different groups of T6SS proposed on phylogenetic grounds by Boyer et al [91]. We performed all against all blast searches, and then clustered genes with MCL (threshold for clustering: e-val < 10^-15^, inflation parameter: I=1.6) [92]. We ended up with 14 gene families found in most of the model T6SS, 13 of them being the most highly conserved according to Boyer et al [91]. Those families corresponded to the following COG families: ‘COG0542', ‘COG3157', ‘COG3455', ‘COG3501', ‘COG3515', ‘COG3516', ‘COG3517', ‘COG3518', ‘COG3519', ‘COG3520', ‘COG3521', ‘COG3522', ‘COG3523', ‘COG4104'. For each of these 14 families, we built sequence profiles with HMMER 3.0 [84] after construction of multiple sequence alignments [93] and manual edition using SEAVIEW [94]. We then performed searches with our profiles in genomic sequences of interest (HMMER 3.0). T6SS were conservatively inferred when at least 12 of the 14 core genes presented contiguous hits (c-value < 10^-3^, criterion for contiguity: core genes can not be separated by more than 15 genes). Our T6SS assignments were consistent with that of Boyer et al. [91], and with data in the literature. For instance, we recovered systems recently described as functional in *B. mallei* [95], *B. pseudomallei* [96], in *P. aeruginosa* [97] and in *Salmonella typhi* [98].

**Identification of T3SS and flagella**. T3SS were identified using protein profiles on 9 genes ubiquitous among these systems: *sctC, sctJ, sctN, sctQ, sctR, sctS, sctT, sctU, sctV* [99] extracted from T3SS of model genomes (profiles were built as for T6SS core genes, see above). The hits were searched using HMMER 3.0 with a local alignment presenting a c-value lower than 1x10^-3^ [84]. As a single cluster (proteobacteria) or several clusters of genes (chlamydiales, [100]) encode T3SS, we searched for clusters of contiguous HMMER hits (no more than 35 genes between contiguous core genes). Eight of these T3SS genes are homologous to core genes of the flagellar system [101], and the two systems were discriminated on the basis of the presence of the T3SS secretin (*sctC*), and absence of three genes exclusive of the flagellum: *flgB*, *flgC* and *fliE*. Clusters of hits containing all the eight core genes, the secretin and no flagellum-specific genes were classified as T3SS, whereas clusters of hits containing the eight core genes but no secretin and at least one of the genes *flgB*, *flgC* and *fliE* were classified as putative flagella. No ambiguous clusters containing both secretin and flagellum-specific genes were recovered, suggesting a good discrimination between the two systems based on our criteria. We made extensive searches in the literature to verify that we were able to retrieve the experimentally verified T3SS, notably in *Yersinia spp.* [102]*, Escherichia coli EPEC* [103]*, Pseudomonas aeruginosa* [104] and *Burkholderia pseudomallei* [105].

**Specific comments**:

***Aeromonas hydrophila*** ATCC 7966. This strain does not have a T3SS [31], but some other isolates have been found to encode one [106]. Given this strain is the type strain and is among the most virulent among *Aeromonas* spp [107], we considered that a T3SS does not make part of the typical repertoire of virulence factors of *Aeromonas hydrophila*. The genome of the strain does code for a T6SS.

***Burkholderia* spp**. Out of 8 strains, one lacked (*Burkholderia mallei* SAVP1) a T3SS. This strain is avirulent after multiple passages in animals possibly as a result of the loss of the T3SS [108].

***Campylobacter jejuni.*** One of the four strains might have a T4SS not linked to conjugation [109], but all others lacked it and we considered that a T4SS does make part of the typical repertoire of virulence factors of the species.

***Mycobacteria*.** While mycobacteria are now often regarded as diderms and not monoderms, there is no known T3SS, T4SS or T6SS dedicated to direct delivery of proteins to eukaryotes in this clade. We have therefore marked them as monoderms along with Firmicutes and Tenericutes only to emphasize this point.

***Shigella***. Most *Shigella* genome sequences do not include the sequence of the accompanying *Shigella* plasmid that encodes the T3SS. We assumed that all *Shigella* had the T3SS, as mentioned in the literature [71,110].

Table S4 – Protein secretion systems data.

| ***species/pathotype*** | ***Diderms*** | ***T3SS*** | ***T4SS*** | ***T6SS*** |
| --- | --- | --- | --- | --- |
| *Aeromonas hydrophila* | 1 | 0 | 0 | 1 |
| *Bacillus anthracis* | 0 | 0 | 0 | 0 |
| *Bacillus cereus* | 0 | 0 | 0 | 0 |
| *Brucella melitensis* | 1 | 0 | 1 | 0 |
| *Burkholderia mallei* | 1 | 1 | 0 | 1 |
| *Burkh. pseudomallei* | 1 | 1 | 0 | 1 |
| *Campylobacter jejuni* | 1 | 0 | 0 | 0 |
| *Clostridium perfringens* | 0 | 0 | 0 | 0 |
| *Coxiella burnetii* | 1 | 0 | 1 | 0 |
| *Enterobacter cloacae* | 1 | 0 | 0 | 0 |
| *E. coli EaggEC* | 1 | 0 | 0 | 1 |
| *E. coli EHEC* | 1 | 1 | 0 | 1 |
| *E. coli EIEC* | 1 | 1 | 0 | 1 |
| *E. coli EPEC* | 1 | 1 | 0 | 1 |
| *E. coli ETEC* | 1 | 0 | 0 | 1 |
| *Enterococcus faecalis* | 0 | 0 | 0 | 0 |
| *Francisella tularensis* | 1 | 0 | 0 | 0 |
| *Gardnerella vaginalis* | 0 | 0 | 0 | 0 |
| *Haemophilus ducreyi* | 1 | 0 | 0 | 0 |
| *Helicobacter pylori* | 1 | 0 | 1 | 0 |
| *Legionella pneumophila* | 1 | 0 | 1 | 0 |
| *Listeria monocytogenes* | 0 | 0 | 0 | 0 |
| *Mycobacterium avium* | 0 | 0 | 0 | 0 |
| *Mycobacterium bovis* | 0 | 0 | 0 | 0 |
| *Mycob. tuberculosis* | 0 | 0 | 0 | 0 |
| *Mycoplasma pneumoniae* | 0 | 0 | 0 | 0 |
| *Neisseria gonorrhoeae* | 1 | 0 | 0 | 0 |
| *Neisseria meningitidis* | 1 | 0 | 0 | 0 |
| *Orientia tsutsugamushi* | 1 | 0 | 1 | 0 |
| *Plesiomonas shigelloides* | 0 | 0 | 0 | 0 |
| *Pseudomonas aeruginosa* | 1 | 1 | 0 | 1 |
| *Rickettsia prowazekii* | 1 | 0 | 1 | 0 |
| *Rickettsia rickettsii* | 1 | 0 | 1 | 0 |
| *S. enterica Typhimurium* | 1 | 1 | 0 | 1 |
| *Salmonella enterica Typhi* | 1 | 1 | 0 | 1 |
| *Shigella dysenteriae* | 1 | 1 | 0 | 1 |
| *Shigella flexneri* | 1 | 1 | 0 | 1 |
| *Shigella sonnei* | 1 | 1 | 0 | 1 |
| *Staphylococcus aureus* | 0 | 0 | 0 | 0 |
| *Stenotrophomonas maltophilia* | 1 | 0 | 0 | 0 |
| *Streptococcus pyogenes* | 0 | 0 | 0 | 0 |
| *Treponema pallidum* | 0 | 0 | 0 | 0 |
| *Vibrio cholerae* | 1 | 0 | 0 | 1 |
| *V. parahaemolyticus* | 1 | 1 | 0 | 1 |
| *Vibrio vulnificus* | 1 | 0 | 0 | 1 |
| *Yersinia enterocolitica* | 1 | 1 | 0 | 1 |
| *Yersinia pestis* | 1 | 1 | 0 | 1 |
| *Y. pseudotuberculosis* | 1 | 1 | 0 | 1 |

# 5. Other traits

Table S5 – Motility (from [145]), minimum generation times (from [146]), and genome size (from Genbank).

| ***species/pathotype*** | ***motility*** | ***d (h)*** | ***genome size (bp)*** |
| --- | --- | --- | --- |
| *Aeromonas hydrophila* | 1 | 0.35 | 4744448 |
| *Bacillus anthracis* | 0 | 0.50 | 5409537 |
| *Bacillus cereus* | 1 | 0.30 | 5409719 |
| *Brucella melitensis* | 0 | 2.00 | 3294819 |
| *Burkholderia mallei* | 0 | 0.75 | 5664653 |
| *Burkh. pseudomallei* | 1 | 1.00 | 7171313 |
| *Campylobacter jejuni* | 1 | 1.10 | 1716752 |
| *Clostridium perfringens* | 0 | 0.20 | 3100837 |
| *Coxiella burnetii* | 0 | 8.00 | 2082121 |
| *Enterobacter cloacae* | 1 | 0.23 | 5206423 |
| *E. coli EaggEC* | 1 | 0.50 | 5154862 |
| *E. coli EHEC* | 1 | 0.33 | 5498450 |
| *E. coli EIEC* | 1 | 0.40 | 5071018 |
| *E. coli EPEC* | 1 | 0.40 | 5202090 |
| *E. coli ETEC* | 1 | 0.33 | 5238056 |
| *Enterococcus faecalis* | 0 | 0.50 | 3359974 |
| *Francisella tularensis* | 0 | 3.00 | 1896307 |
| *Gardnerella vaginalis* | 0 | 13.50 | 1642448 |
| *Haemophilus ducreyi* | 1 | 1.80 | 1698955 |
| *Helicobacter pylori* | 1 | 2.40 | 1642614 |
| *Legionella pneumophila* | 1 | 3.30 | 3506314 |
| *Listeria monocytogenes* | 1(0) | 1.00 | 2974502 |
| *Mycobacterium avium* | 0 | 10.00 | 5152636 |
| *Mycobacterium bovis* | 0 | 23.00 | 4363908 |
| *Mycob. tuberculosis* | 0 | 19.00 | 4411606 |
| *Mycoplasma pneumoniae* | 1 | 6.00 | 816394 |
| *Neisseria gonorrhoeae* | 0 | 0.58 | 2195050 |
| *Neisseria meningitidis* | 0 | 0.72 | 2184406 |
| *Orientia tsutsugamushi* | 0 | 9.00 | 2070096 |
| *Plesiomonas shigelloides* | 1 | 0.43 | . |
| *Pseudomonas aeruginosa* | 1 | 0.50 | 6498037 |
| *Rickettsia prowazekii* | 0 | 10.00 | 1111523 |
| *Rickettsia rickettsii* | 0 | 9.00 | 1262943 |
| *S. enterica Typhimurium* | 1 | 0.40 | 4857432 |
| *Salmonella enterica Typhi* | 1 | 0.40 | 4809037 |
| *Shigella dysenteriae* | 0 | 0.67 | 4369232 |
| *Shigella flexneri* | 0 | 0.68 | 4828821 |
| *Shigella sonnei* | 0 | 0.50 | 4825265 |
| *Staphylococcus aureus* | 0 | 0.40 | 2855443 |
| *Stenotrophomonas maltophilia* | 1 | 0.60 | 4851126 |
| *Streptococcus pyogenes* | 0 | 0.40 | 1876731 |
| *Treponema pallidum* | 1 | 33.00 | 1138734 |
| *Vibrio cholerae* | 1 | 0.20 | 4085264 |
| *V. parahaemolyticus* | 1 | 0.20 | 5165770 |
| *Vibrio vulnificus* | 1 | 0.16 | 5131550 |
| *Yersinia enterocolitica* | 1 | 0.55 | 4652716 |
| *Yersinia pestis* | 0 | 1.25 | 4803663 |
| *Y. pseudotuberculosis* | 1(0) | 0.50 | 4845396 |

# 6. Quorum sensing

Table S6- Data on quorum-sensing. The literature was mined for the presence/absence of response to quorum sensing (column QS) and then to the regulatory dependence of virulence factors upon QS (column V-QS). Only the latter is used in the text.

| ***species/pathotype*** | ***QS*** | ***V-QS*** | ***References/comments*** |
| --- | --- | --- | --- |
| *Aeromonas hydrophila* | Y | Y | [111] |
| *Bacillus anthracis* | Y | Y | [112] |
| *Bacillus cereus* | Y | Y | [113] |
| *Brucella melitensis* | Y | Y | [114] |
| *Burkholderia mallei* | Y | Y | [115] |
| *Burkh. pseudomallei* | Y | Y | [116] |
| *Campylobacter jejuni* | Y | N | Published evidence that QS is implicated in stress response and colonization of chickens, but no evidence of implication in virulence in humans [117]. |
| *Clostridium perfringens* | Y | Y | [118] |
| *Coxiella burnetii* | N | N | Group V isolates contained 2 ORFs homologs to luxR. The lack of additional ORFs related to QS within the genome suggests they serve other regulatory functions [119]. |
| *Enterobacter cloacae* | N | N |  |
| *E. coli EaggEC* | Y | Y | QS is putatively involved in aggregation behavior which is a virulence trait in E. coli EaggEC [120]. |
| *E. coli EHEC* | Y | Y | [121]. |
| *E. coli EIEC* | Y | N | See S. sonnei comment [122] |
| *E. coli EPEC* | Y | Y | [121]. |
| *E. coli ETEC* | Y | Y | [123] |
| *Enterococcus faecalis* | Y | Y | [124] |
| *Francisella tularensis* | Y | Y | [125] |
| *Gardnerella vaginalis* | Y | Y | [126] |
| *Haemophilus ducreyi* | N | N |  |
| *Helicobacter pylori* | Y | N | [127] |
| *Legionella pneumophila* | Y | Y | [128] |
| *Listeria monocytogenes* | Y | Y | [129] |
| *Mycobacterium avium* | N | N | While it has been proposed that QS regulates virulence in M. avium, recent data is not in accordance with this view [130]. |
| *Mycobacterium bovis* | N | N |  |
| *Mycob. tuberculosis* | N | N | Expression patterns are correlated with bacterial density [131], but no QS described in the literature. |
| *Mycoplasma pneumoniae* | N | N |  |
| *Neisseria gonorrhoeae* | N | N |  |
| *Neisseria meningitidis* | N | N | While there is expression of AI-2 by *N. meningitidis*, evidence suggests this is a metabolic by-product and not used as a cell-to-cell signaling molecule [132] |
| *Orientia tsutsugamushi* | N | N |  |
| *Plesiomonas shigelloides* | N | N | No AHL signals detected [133]. |
| *Pseudomonas aeruginosa* | Y | Y | [134] |
| *Rickettsia prowazekii* | N | N |  |
| *Rickettsia rickettsii* | N | N |  |
| *S. enterica Typhimurium* | Y | Y | [135] |
| *Salmonella enterica Typhi* | Y | N | QS is known to be relevant for carriage (biofilm formation), but no evidence for its relevance in infection [136]. |
| *Shigella dysenteriae* | Y | N | See S. sonnei comment [122] |
| *Shigella flexneri* | Y | N | See S. sonnei comment [122] |
| *Shigella sonnei* | Y | N | The AI-2 signaling molecule does not influence invasion operon expression and is not required for Shigella or EIEC virulence, as mutants deficient in AI-2 synthesis are fully virulent [122]. |
| *Staphylococcus aureus* | Y | Y | [137] |
| *Stenotrophomonas maltophilia* | Y | Y | [138] |
| *Streptococcus pyogenes* | Y | Y | [139] |
| *Treponema pallidum* | N | N |  |
| *Vibrio cholerae* | Y | Y | [140] |
| *V. parahaemolyticus* | Y | Y | [141] |
| *Vibrio vulnificus* | Y | Y | [142] |
| *Yersinia enterocolitica* | Y | Y | QS regulates the T3SS which is a virulence factor [143]. |
| *Yersinia pestis* | Y | Y | [144] |
| *Y. pseudotuberculosis* | Y | Y | QS regulates the T3SS which is a virulence factor [143]. |

# References

1. Solnick JV, Hansen LM, Canfield DR, Parsonnet J (2001) Determination of the infectious dose of Helicobacter pylori during primary and secondary infection in rhesus monkeys (Macaca mulatta). Infect Immun 69: 6887-6892.

2. Ferrero RL, Thiberge JM, Kansau I, Wuscher N, Huerre M, et al. (1995) The GroES homolog of Helicobacter pylori confers protective immunity against mucosal infection in mice. Proc Natl Acad Sci U S A 92: 6499-6503.

3. Feldman RA, Eccersley AJ, Hardie JM (1998) Epidemiology of Helicobacter pylori: acquisition, transmission, population prevalence and disease-to-infection ratio. Br Med Bull 54: 39-53.

4. Rusin PA, Rose JB, Haas CN, Gerba CP (1997) Risk assessment of opportunistic bacterial pathogens in drinking water. Rev Environ Contam Toxicol 152: 57-83.

5. Glasmacher A, Engelherat S, Exner M (2003) Infections from HCP organisms in drinking-water amongst the immunocompromised. . In: Bartram J, Cotruvo, J., Exner, M., Fricker, F., Glasmacher, A. , editor. Heterotrophic Plate Counts and Drinking- water Safety. London: IWA. pp. pp. 137-145.

6. Muller D, Edwards ML, Smith DW (1983) Changes in iron and transferrin levels and body temperature in experimental airborne legionellosis. J Infect Dis 147: 302-307.

7. Berendt RF, Young HW, Allen RG, Knutsen GL (1980) Dose-response of guinea pigs experimentally infected with aerosols of Legionella pneumophila. J Infect Dis 141: 186-192.

8. Armstrong TW, Haas CN (2007) A quantitative microbial risk assessment model for Legionnaires' disease: animal model selection and dose-response modeling. Risk Anal 27: 1581-1596.

9. Addiss DG, Davis JP, LaVenture M, Wand PJ, Hutchinson MA, et al. (1989) Community-acquired Legionnaires' disease associated with a cooling tower: evidence for longer-distance transport of Legionella pneumophila. Am J Epidemiol 130: 557-568.

10. Engelhart S, Pleischl S, Luck C, Marklein G, Fischnaller E, et al. (2008) Hospital-acquired legionellosis originating from a cooling tower during a period of thermal inversion. Int J Hyg Environ Health 211: 235-240.

11. CDC (2011).

12. Tuanyok A, Tom M, Dunbar J, Woods DE (2006) Genome-wide expression analysis of Burkholderia pseudomallei infection in a hamster model of acute melioidosis. Infect Immun 74: 5465-5476.

13. Sewell DL (1995) Laboratory-associated infections and biosafety. Clin Microbiol Rev 8: 389-405.

14. Cash RA, Music SI, Libonati JP, Snyder MJ, Wenzel RP, et al. (1974) Response of man to infection with Vibrio cholerae. I. Clinical, serologic, and bacteriologic responses to a known inoculum. J Infect Dis 129: 45-52.

15. CFSAN (2011) FDA bad bug book.

16. HCW (2011).

17. Barceloux DG (2008) Medical toxicology of natural substances: Foods, Fungi, Medicinal Herbs, Plants, and Venomous Animals. Hoboken, NJ: John Wiley & Sons. 1180 p.

18. McCullough NB, Eisele CW (1951) Experimental human salmonellosis. III. Pathogenicity of strains of Salmonella newport, Salmonella derby, and Salmonella bareilly obtained from spray-dried whole egg. J Infect Dis 89: 209-213.

19. McCullough NB, Eisele CW (1951) Experimental human salmonellosis. I. Pathogenicity of strains of Salmonella meleagridis and Salmonella anatum obtained from spray-dried whole egg. J Infect Dis 88: 278-289.

20. Hook EW (1961) Salmonellosis: certain factors influencing the interaction of Salmonella and the human host. Bull N Y Acad Med 37: 499-512.

21. Kothary MH, Babu US (2001) Infective dose of foodborne pathogens in volunteers: a review. J Food Safety 21: 49-73.

22. Greenwood MH, Hooper WL (1983) Chocolate bars contaminated with Salmonella napoli: an infectivity study. Br Med J (Clin Res Ed) 286: 1394.

23. Woods JB (2005) USAMRIID’s Medical management of biological casualties handbook. Frederick, Maryland: U.S. Army Medical Research. 187 p.

24. Propst KL, Mima T, Choi KH, Dow SW, Schweizer HP (2010) A Burkholderia pseudomallei deltapurM mutant is avirulent in immunocompetent and immunodeficient animals: candidate strain for exclusion from select-agent lists. Infect Immun 78: 3136-3143.

25. Criswell BS, Ladwig CL, Gardner HL, Dukes CD (1969) Haemophilus vaginalis: vaginitis by inoculation from culture. Obstet Gynecol 33: 195-199.

26. Schaad UB (1983) Which number of infecting bacteria is of clinical relevance? Infection 11 Suppl 2: S87-89.

27. Schneider H, Cross AS, Kuschner RA, Taylor DN, Sadoff JC, et al. (1995) Experimental human gonococcal urethritis: 250 Neisseria gonorrhoeae MS11mkC are infective. J Infect Dis 172: 180-185.

28. Elek SD, Conen PE (1957) The virulence of Staphylococcus pyogenes for man; a study of the problems of wound infection. Br J Exp Pathol 38: 573-586.

29. Kashimoto T, Ueno S, Hanajima M, Hayashi H, Akeda Y, et al. (2003) Vibrio vulnificus induces macrophage apoptosis in vitro and in vivo. Infect Immun 71: 533-535.

30. Areschoug T, Carlsson F, Stalhammar-Carlemalm M, Lindahl G (2004) Host-pathogen interactions in Streptococcus pyogenes infections, with special reference to puerperal fever and a comment on vaccine development. Vaccine 22 Suppl 1: S9-S14.

31. Seshadri R, Joseph SW, Chopra AK, Sha J, Shaw J, et al. (2006) Genome sequence of Aeromonas hydrophila ATCC 7966T: jack of all trades. J Bacteriol 188: 8272-8282.

32. Baldari CT, Tonello F, Paccani SR, Montecucco C (2006) Anthrax toxins: A paradigm of bacterial immune suppression. Trends Immunol 27: 434-440.

33. Rasko DA, Altherr MR, Han CS, Ravel J (2005) Genomics of the Bacillus cereus group of organisms. FEMS Microbiol Rev 29: 303-329.

34. Gorvel JP (2008) Brucella: a Mr "Hide" converted into Dr Jekyll. Microbes Infect 10: 1010-1013.

35. Galyov EE, Brett PJ, DeShazer D (2010) Molecular insights into Burkholderia pseudomallei and Burkholderia mallei pathogenesis. Annu Rev Microbiol 64: 495-517.

36. Ribot WJ, Ulrich RL (2006) The animal pathogen-like type III secretion system is required for the intracellular survival of Burkholderia mallei within J774.2 macrophages. Infect Immun 74: 4349-4353.

37. Kiehlbauch JA, Albach RA, Baum LL, Chang KP (1985) Phagocytosis of Campylobacter jejuni and its intracellular survival in mononuclear phagocytes. Infect Immun 48: 446-451.

38. Hickey TE, Majam G, Guerry P (2005) Intracellular survival of Campylobacter jejuni in human monocytic cells and induction of apoptotic death by cytholethal distending toxin. Infect Immun 73: 5194-5197.

39. O'Brien DK, Melville SB (2004) Effects of Clostridium perfringens alpha-toxin (PLC) and perfringolysin O (PFO) on cytotoxicity to macrophages, on escape from the phagosomes of macrophages, and on persistence of C. perfringens in host tissues. Infect Immun 72: 5204-5215.

40. Capo C, Lindberg FP, Meconi S, Zaffran Y, Tardei G, et al. (1999) Subversion of monocyte functions by coxiella burnetii: impairment of the cross-talk between alphavbeta3 integrin and CR3. J Immunol 163: 6078-6085.

41. Krzyminska S, Mokracka J, Koczura R, Kaznowski A (2009) Cytotoxic activity of Enterobacter cloacae human isolates. FEMS Immunol Med Microbiol 56: 248-252.

42. Healy B, Cooney S, O'Brien S, Iversen C, Whyte P, et al. (2010) Cronobacter (Enterobacter sakazakii): an opportunistic foodborne pathogen. Foodborne Pathog Dis 7: 339-350.

43. Kaper JB, Nataro JP, Mobley HL (2004) Pathogenic Escherichia coli. Nat Rev Microbiol 2: 123-140.

44. Poirier K, Faucher SP, Beland M, Brousseau R, Gannon V, et al. (2008) Escherichia coli O157:H7 survives within human macrophages: global gene expression profile and involvement of the Shiga toxins. Infect Immun 76: 4814-4822.

45. Celli J, Olivier M, Finlay BB (2001) Enteropathogenic Escherichia coli mediates antiphagocytosis through the inhibition of PI 3-kinase-dependent pathways. Embo J 20: 1245-1258.

46. Byrd W, Mog SR, Cassels FJ (2003) Pathogenicity and immune response measured in mice following intranasal challenge with enterotoxigenic Escherichia coli strains H10407 and B7A. Infect Immun 71: 13-21.

47. Giard JC, Riboulet E, Verneuil N, Sanguinetti M, Auffray Y, et al. (2006) Characterization of Ers, a PrfA-like regulator of Enterococcus faecalis. FEMS Immunol Med Microbiol 46: 410-418.

48. Lofgren S, Tarnvik A, Bloom GD, Sjoberg W (1983) Phagocytosis and killing of Francisella tularensis by human polymorphonuclear leukocytes. Infect Immun 39: 715-720.

49. Sjostedt A (2006) Intracellular survival mechanisms of Francisella tularensis, a stealth pathogen. Microbes Infect 8: 561-567.

50. Easmon CS, Clark L, Crane JP, Green R (1985) Phagocytosis and killing of Gardnerella vaginalis by human neutrophils. J Clin Pathol 38: 747-749.

51. Ahmed HJ, Johansson C, Svensson LA, Ahlman K, Verdrengh M, et al. (2002) In vitro and in vivo interactions of Haemophilus ducreyi with host phagocytes. Infect Immun 70: 899-908.

52. Xu T, Lundqvist A, Ahmed HJ, Eriksson K, Yang Y, et al. (2004) Interactions of Haemophilus ducreyi and purified cytolethal distending toxin with human monocyte-derived dendritic cells, macrophages and CD4+ T cells. Microbes Infect 6: 1171-1181.

53. Wilson KT, Crabtree JE (2007) Immunology of Helicobacter pylori: insights into the failure of the immune response and perspectives on vaccine studies. Gastroenterology 133: 288-308.

54. Gebert B, Fischer W, Weiss E, Hoffmann R, Haas R (2003) Helicobacter pylori vacuolating cytotoxin inhibits T lymphocyte activation. Science 301: 1099-1102.

55. Borlace GN, Jones HF, Keep SJ, Butler RN, Brooks DA (2011) Helicobacter pylori phagosome maturation in primary human macrophages. Gut Pathog 3: 3.

56. Bellinger-Kawahara C, Horwitz MA (1990) Complement component C3 fixes selectively to the major outer membrane protein (MOMP) of Legionella pneumophila and mediates phagocytosis of liposome-MOMP complexes by human monocytes. J Exp Med 172: 1201-1210.

57. Vogel J, Andrews H, Wong S, Isberg R (1998) Conjugative transfer by the virulence system of Legionella pneumophila. Science 279: 873-876.

58. Vazquez-Boland JA, Kuhn M, Berche P, Chakraborty T, Dominguez-Bernal G, et al. (2001) Listeria pathogenesis and molecular virulence determinants. Clin Microbiol Rev 14: 584-640.

59. Sturgill-Koszycki S, Schlesinger PH, Chakraborty P, Haddix PL, Collins HL, et al. (1994) Lack of acidification in Mycobacterium phagosomes produced by exclusion of the vesicular proton-ATPase. Science 263: 678-681.

60. Flynn JL, Chan J (2001) Immunology of tuberculosis. Annu Rev Immunol 19: 93-129.

61. Stenger S, Niazi KR, Modlin RL (1998) Down-regulation of CD1 on antigen-presenting cells by infection with Mycobacterium tuberculosis. J Immunol 161: 3582-3588.

62. Shafer WM, Rest RF (1989) Interactions of gonococci with phagocytic cells. Annu Rev Microbiol 43: 121-145.

63. Franzoso S, Mazzon C, Sztukowska M, Cecchini P, Kasic T, et al. (2008) Human monocytes/macrophages are a target of Neisseria meningitidis Adhesin A (NadA). J Leukoc Biol 83: 1100-1110.

64. Rikihisa Y, Ito S (1979) Intracellular localization of Rickettsia tsutsugamushi in polymorphonuclear leukocytes. J Exp Med 150: 703-708.

65. Seong SY, Choi MS, Kim IS (2001) Orientia tsutsugamushi infection: overview and immune responses. Microbes Infect 3: 11-21.

66. Lau GW, Hassett DJ, Britigan BE (2005) Modulation of lung epithelial functions by Pseudomonas aeruginosa. Trends Microbiol 13: 389-397.

67. Rosenberger CM, Finlay BB (2003) Phagocyte sabotage: disruption of macrophage signalling by bacterial pathogens. Nat Rev Mol Cell Biol 4: 385-396.

68. Weiss E (1982) The biology of rickettsiae. Annu Rev Microbiol 36: 345-370.

69. Monack DM, Raupach B, Hromockyj AE, Falkow S (1996) Salmonella typhimurium invasion induces apoptosis in infected macrophages. Proc Natl Acad Sci U S A 93: 9833-9838.

70. Jantsch J, Chikkaballi D, Hensel M (2011) Cellular aspects of immunity to intracellular Salmonella enterica. Immunol Rev 240: 185-195.

71. Schroeder GN, Hilbi H (2008) Molecular pathogenesis of Shigella spp.: controlling host cell signaling, invasion, and death by type III secretion. Clin Microbiol Rev 21: 134-156.

72. Kobayashi SD, Braughton KR, Palazzolo-Ballance AM, Kennedy AD, Sampaio E, et al. (2010) Rapid neutrophil destruction following phagocytosis of Staphylococcus aureus. J Innate Immun 2: 560-575.

73. Waters VJ, Gomez MI, Soong G, Amin S, Ernst RK, et al. (2007) Immunostimulatory properties of the emerging pathogen Stenotrophomonas maltophilia. Infect Immun 75: 1698-1703.

74. Osterlund A, Popa R, Nikkila T, Scheynius A, Engstrand L (1997) Intracellular reservoir of Streptococcus pyogenes in vivo: a possible explanation for recurrent pharyngotonsillitis. Laryngoscope 107: 640-647.

75. Radolf JD, Desrosiers DC (2009) Treponema pallidum, the stealth pathogen, changes, but how? Mol Microbiol 72: 1081-1086.

76. Lukehart SA, Shaffer JM, Baker-Zander SA (1992) A subpopulation of Treponema pallidum is resistant to phagocytosis: possible mechanism of persistence. J Infect Dis 166: 1449-1453.

77. Juanpere-Rodero N, Martin-Ezquerra G, Fernandez-Casado A, Magan-Perea L, Garcia-Alguacil MA, et al. (2011) Cell and Tissue Interactions of Treponema pallidum in Primary and Secondary Syphilitic Skin Lesions: An Ultrastructural Study of Serial Sections. Ultrastruct Pathol.

78. Baker-Zander SA, Shaffer JM, Lukehart SA (1993) Characterization of the serum requirement for macrophage-mediated killing of Treponema pallidum ssp. pallidum: relationship to the development of opsonizing antibodies. FEMS Immunol Med Microbiol 6: 273-279.

79. Mekalanos JJ (1983) Duplication and amplification of toxin genes in Vibrio cholerae. Cell 35: 253-263.

80. Broberg CA, Calder TJ, Orth K (2011) Vibrio parahaemolyticus cell biology and pathogenicity determinants. Microbes Infect.

81. Horseman MA, Surani S (2011) A comprehensive review of Vibrio vulnificus: an important cause of severe sepsis and skin and soft-tissue infection. Int J Infect Dis 15: e157-166.

82. Cornelis GR, Boland A, Boyd AP, Geuijen C, Iriarte M, et al. (1998) The virulence plasmid of Yersinia, an antihost genome. Microbiol Mol Biol Rev 62: 1315-1352.

83. Smillie C, Garcillan-Barcia MP, Francia MV, Rocha EP, de la Cruz F (2010) Mobility of plasmids. Microbiol Mol Biol Rev 74: 434-452.

84. Eddy SR (1998) Profile hidden Markov models. Bioinformatics 14: 755-763.

85. Alvarez-Martinez CE, Christie PJ (2009) Biological diversity of prokaryotic type IV secretion systems. Microbiol Mol Biol Rev 73: 775-808.

86. de la Cruz F, Frost LS, Meyer RJ, Zechner E (2010) Conjugative DNA Metabolism in Gram-negative Bacteria. FEMS Microbiol Rev 34: 18-40.

87. Boschiroli ML, Ouahrani-Bettache S, Foulongne V, Michaux-Charachon S, Bourg G, et al. (2002) Type IV secretion and Brucella virulence. Vet Microbiol 90: 341-348.

88. Seshadri R, Paulsen IT, Eisen JA, Read TD, Nelson KE, et al. (2003) Complete genome sequence of the Q-fever pathogen Coxiella burnetii. Proc Natl Acad Sci U S A 100: 5455-5460.

89. Censini S, Lange C, Xiang Z, Crabtree JE, Ghiara P, et al. (1996) cag, a pathogenicity island of Helicobacter pylori, encodes type I-specific and disease-associated virulence factors. Proc Natl Acad Sci U S A 93: 14648-14653.

90. Rikihisa Y, Lin M (2010) Anaplasma phagocytophilum and Ehrlichia chaffeensis type IV secretion and Ank proteins. Curr Opin Microbiol 13: 59-66.

91. Boyer F, Fichant G, Berthod J, Vandenbrouck Y, Attree I (2009) Dissecting the bacterial type VI secretion system by a genome wide in silico analysis: what can be learned from available microbial genomic resources? BMC Genomics 10: 104.

92. Enright AJ, Van Dongen S, Ouzounis CA (2002) An efficient algorithm for large-scale detection of protein families. Nucleic Acids Res 30: 1575-1584.

93. Edgar RC (2004) MUSCLE: multiple sequence alignment with high accuracy and high throughput. Nucleic Acids Res 32: 1792-1797.

94. Gouy M, Guindon S, Gascuel O (2010) SeaView version 4: A multiplatform graphical user interface for sequence alignment and phylogenetic tree building. Mol Biol Evol 27: 221-224.

95. Schell MA, Ulrich RL, Ribot WJ, Brueggemann EE, Hines HB, et al. (2007) Type VI secretion is a major virulence determinant in Burkholderia mallei. Mol Microbiol 64: 1466-1485.

96. Burtnick MN, Brett PJ, Harding SV, Ngugi SA, Ribot WJ, et al. (2011) The cluster 1 type VI secretion system is a major virulence determinant in Burkholderia pseudomallei. Infect Immun 79: 1512-1525.

97. Mougous JD, Cuff ME, Raunser S, Shen A, Zhou M, et al. (2006) A virulence locus of Pseudomonas aeruginosa encodes a protein secretion apparatus. Science 312: 1526-1530.

98. Wang M, Luo Z, Du H, Xu S, Ni B, et al. (2011) Molecular characterization of a functional type VI secretion system in Salmonella enterica serovar Typhi. Curr Microbiol 63: 22-31.

99. Cornelis GR (2006) The type III secretion injectisome. Nat Rev Microbiol 4: 811-825.

100. Kim JF (2001) Revisiting the chlamydial type III protein secretion system: clues to the origin of type III protein secretion. Trends Genet 17: 65-69.

101. Pallen MJ, Beatson SA, Bailey CM (2005) Bioinformatics, genomics and evolution of non-flagellar type-III secretion systems: a Darwinian perspective. FEMS Microbiol Rev 29: 201-229.

102. Cornelis GR, Wolf-Watz H (1997) The Yersinia Yop virulon: a bacterial system for subverting eukaryotic cells. Mol Microbiol 23: 861-867.

103. Jarvis KG, Giron JA, Jerse AE, McDaniel TK, Donnenberg MS, et al. (1995) Enteropathogenic Escherichia coli contains a putative type III secretion system necessary for the export of proteins involved in attaching and effacing lesion formation. Proc Natl Acad Sci U S A 92: 7996-8000.

104. Yahr TL, Goranson J, Frank DW (1996) Exoenzyme S of Pseudomonas aeruginosa is secreted by a type III pathway. Mol Microbiol 22: 991-1003.

105. Stevens MP, Wood MW, Taylor LA, Monaghan P, Hawes P, et al. (2002) An Inv/Mxi-Spa-like type III protein secretion system in Burkholderia pseudomallei modulates intracellular behaviour of the pathogen. Mol Microbiol 46: 649-659.

106. Sha J, Pillai L, Fadl AA, Galindo CL, Erova TE, et al. (2005) The type III secretion system and cytotoxic enterotoxin alter the virulence of Aeromonas hydrophila. Infect Immun 73: 6446-6457.

107. Janda JM, Kokka RP (1991) The pathogenicity of Aeromonas strains relative to genospecies and phenospecies identification. FEMS Microbiol Lett 69: 29-33.

108. Schutzer SE, Schlater LR, Ronning CM, DeShazer D, Luft BJ, et al. (2008) Characterization of clinically-attenuated Burkholderia mallei by whole genome sequencing: candidate strain for exclusion from Select Agent lists. PLoS ONE 3: e2058.

109. Guglielmini J, Quintais L, Garcillan-Barcia MP, de la Cruz F, Rocha EP (2011) The Repertoire of ICE in Prokaryotes Underscores the Unity, Diversity, and Ubiquity of Conjugation. PLoS Genet 7: e1002222.

110. Buchrieser C, Glaser P, Rusniok C, Nedjari H, D'Hauteville H, et al. (2000) The virulence plasmid pWR100 and the repertoire of proteins secreted by the type III secretion apparatus of shigella flexneri. Mol Microbiol 38: 760-771.

111. Swift S, Lynch MJ, Fish L, Kirke DF, Tomas JM, et al. (1999) Quorum sensing-dependent regulation and blockade of exoprotease production in Aeromonas hydrophila. Infect Immun 67: 5192-5199.

112. Jones MB, Peterson SN, Benn R, Braisted JC, Jarrahi B, et al. (2010) Role of luxS in Bacillus anthracis growth and virulence factor expression. Virulence 1: 72-83.

113. Declerck N, Bouillaut L, Chaix D, Rugani N, Slamti L, et al. (2007) Structure of PlcR: Insights into virulence regulation and evolution of quorum sensing in Gram-positive bacteria. Proc Natl Acad Sci U S A 104: 18490-18495.

114. Rambow-Larsen AA, Rajashekara G, Petersen E, Splitter G (2008) Putative quorum-sensing regulator BlxR of Brucella melitensis regulates virulence factors including the type IV secretion system and flagella. J Bacteriol 190: 3274-3282.

115. Ulrich RL, Deshazer D, Hines HB, Jeddeloh JA (2004) Quorum sensing: a transcriptional regulatory system involved in the pathogenicity of Burkholderia mallei. Infect Immun 72: 6589-6596.

116. Williams P, Winzer K, Chan WC, Camara M (2007) Look who's talking: communication and quorum sensing in the bacterial world. Philos Trans R Soc Lond B Biol Sci 362: 1119-1134.

117. Murphy C, Carroll C, Jordan KN (2006) Environmental survival mechanisms of the foodborne pathogen Campylobacter jejuni. J Appl Microbiol 100: 623-632.

118. Cheung JK, Keyburn AL, Carter GP, Lanckriet AL, Van Immerseel F, et al. (2010) The VirSR two-component signal transduction system regulates NetB toxin production in Clostridium perfringens. Infect Immun 78: 3064-3072.

119. Beare PA, Samuel JE, Howe D, Virtaneva K, Porcella SF, et al. (2006) Genetic diversity of the Q fever agent, Coxiella burnetii, assessed by microarray-based whole-genome comparisons. J Bacteriol 188: 2309-2324.

120. Imuta N, Nishi J, Tokuda K, Fujiyama R, Manago K, et al. (2008) The Escherichia coli efflux pump TolC promotes aggregation of enteroaggregative E. coli 042. Infect Immun 76: 1247-1256.

121. Sperandio V, Li CC, Kaper JB (2002) Quorum-sensing Escherichia coli regulator A: a regulator of the LysR family involved in the regulation of the locus of enterocyte effacement pathogenicity island in enterohemorrhagic E. coli. Infect Immun 70: 3085-3093.

122. Day WA, Jr., Maurelli AT (2001) Shigella flexneri LuxS quorum-sensing system modulates virB expression but is not essential for virulence. Infect Immun 69: 15-23.

123. Zhu J, Yin X, Yu H, Zhao L, Sabour P, et al. (2011) Involvement of quorum sensing and heat-stable enterotoxin a in cell damage caused by a porcine enterotoxigenic Escherichia coli strain. Infect Immun 79: 1688-1695.

124. Haas W, Shepard BD, Gilmore MS (2002) Two-component regulator of Enterococcus faecalis cytolysin responds to quorum-sensing autoinduction. Nature 415: 84-87.

125. Mokrievich AN, Kondakova AN, Valade E, Platonov ME, Vakhrameeva GM, et al. (2010) Biological properties and structure of the lipopolysaccharide of a vaccine strain of Francisella tularensis generated by inactivation of a quorum sensing system gene qseC. Biochemistry (Mosc) 75: 443-451.

126. Verstraelen H (2008) Cutting edge: the vaginal microflora and bacterial vaginosis. Verh K Acad Geneeskd Belg 70: 147-174.

127. Rader BA, Campagna SR, Semmelhack MF, Bassler BL, Guillemin K (2007) The quorum-sensing molecule autoinducer 2 regulates motility and flagellar morphogenesis in Helicobacter pylori. J Bacteriol 189: 6109-6117.

128. Spirig T, Tiaden A, Kiefer P, Buchrieser C, Vorholt JA, et al. (2008) The Legionella autoinducer synthase LqsA produces an alpha-hydroxyketone signaling molecule. J Biol Chem 283: 18113-18123.

129. Riedel CU, Monk IR, Casey PG, Waidmann MS, Gahan CG, et al. (2009) AgrD-dependent quorum sensing affects biofilm formation, invasion, virulence and global gene expression profiles in Listeria monocytogenes. Mol Microbiol 71: 1177-1189.

130. Geier H, Mostowy S, Cangelosi GA, Behr MA, Ford TE (2008) Autoinducer-2 triggers the oxidative stress response in Mycobacterium avium, leading to biofilm formation. Appl Environ Microbiol 74: 1798-1804.

131. Banaiee N, Jacobs WR, Jr., Ernst JD (2006) Regulation of Mycobacterium tuberculosis whiB3 in the mouse lung and macrophages. Infect Immun 74: 6449-6457.

132. Dove JE, Yasukawa K, Tinsley CR, Nassif X (2003) Production of the signalling molecule, autoinducer-2, by Neisseria meningitidis: lack of evidence for a concerted transcriptional response. Microbiology 149: 1859-1869.

133. Ciznar I, Hostacka A, Gonzalez-Rey C, Krovacek K (2004) Potential virulence-associated properties of Plesiomonas shigelloides strains. Folia Microbiol (Praha) 49: 543-548.

134. Winson MK, Camara M, Latifi A, Foglino M, Chhabra SR, et al. (1995) Multiple N-acyl-L-homoserine lactone signal molecules regulate production of virulence determinants and secondary metabolites in Pseudomonas aeruginosa. Proc Natl Acad Sci U S A 92: 9427-9431.

135. Soares JA, Ahmer BM (2011) Detection of acyl-homoserine lactones by Escherichia and Salmonella. Curr Opin Microbiol 14: 188-193.

136. Prouty AM, Schwesinger WH, Gunn JS (2002) Biofilm formation and interaction with the surfaces of gallstones by Salmonella spp. Infect Immun 70: 2640-2649.

137. Novick RP, Geisinger E (2008) Quorum sensing in staphylococci. Annu Rev Genet 42: 541-564.

138. Fouhy Y, Scanlon K, Schouest K, Spillane C, Crossman L, et al. (2007) Diffusible signal factor-dependent cell-cell signaling and virulence in the nosocomial pathogen Stenotrophomonas maltophilia. J Bacteriol 189: 4964-4968.

139. Lyon WR, Madden JC, Levin JC, Stein JL, Caparon MG (2001) Mutation of luxS affects growth and virulence factor expression in Streptococcus pyogenes. Mol Microbiol 42: 145-157.

140. Miller MB, Skorupski K, Lenz DH, Taylor RK, Bassler BL (2002) Parallel quorum sensing systems converge to regulate virulence in Vibrio cholerae. Cell 110: 303-314.

141. Defoirdt T, Crab R, Wood TK, Sorgeloos P, Verstraete W, et al. (2006) Quorum sensing-disrupting brominated furanones protect the gnotobiotic brine shrimp Artemia franciscana from pathogenic Vibrio harveyi, Vibrio campbellii, and Vibrio parahaemolyticus isolates. Appl Environ Microbiol 72: 6419-6423.

142. Kim SY, Lee SE, Kim YR, Kim CM, Ryu PY, et al. (2003) Regulation of Vibrio vulnificus virulence by the LuxS quorum-sensing system. Mol Microbiol 48: 1647-1664.

143. Atkinson S, Goldstone RJ, Joshua GW, Chang CY, Patrick HL, et al. (2011) Biofilm development on Caenorhabditis elegans by Yersinia is facilitated by quorum sensing-dependent repression of type III secretion. PLoS Pathog 7: e1001250.

144. Gelhaus HC, Rozak DA, Nierman WC, Chen D, Varga JJ, et al. (2009) Exogenous Yersinia pestis quorum sensing molecules N-octanoyl-homoserine lactone and N-(3-oxooctanoyl)-homoserine lactone regulate the LcrV virulence factor. Microb Pathog 46: 283-287.

145. Brenner DJ, Krieg NR, Staley JT (2005) Bergey's Manual of Systematic Bacteriology; Garrity G, editor: Springer. 2816 p.

146. Vieira-Silva S, Rocha EPC (2010) The Systemic Imprint of Growth and its Uses in Ecological (Meta)genomics. PLoS Genet 6: e1000808.
